# A 2,000-year Bayesian NAO reconstruction from the Iberian Peninsula

**DOI:** 10.1038/s41598-020-71372-5

**Published:** 2020-09-11

**Authors:** Armand Hernández, Guiomar Sánchez-López, Sergi Pla-Rabes, Laia Comas-Bru, Andrew Parnell, Niamh Cahill, Adelina Geyer, Ricardo M. Trigo, Santiago Giralt

**Affiliations:** 1 Geosciences Barcelona (GEO3BCN-CSIC), Barcelona, Spain; 2 CREAF, Campus de Bellaterra (UAB), Edifici C, 08193 Cerdanyola del Vallès, Spain; 3 School of Archaeology, Geography and Environmental Sciences, University of Reading, Reading, UK; 4 Hamilton Institute, Insight Centre for Data Analytics, Maynooth University, Kildare, Ireland; 5 Department of Mathematics and Statistics, Maynooth University, Maynooth, Kildare, Ireland; 6 Instituto Dom Luiz (IDL), Faculdade de Ciências, Universidade de Lisboa, 1749-016 Lisbon, Portugal; 7 Departamento de Meteorologia, Universidade Federal Do Rio de Janeiro, Rio de Janeiro, 21941-916 Brazil

**Keywords:** Climate sciences, Palaeoclimate

## Abstract

The North Atlantic Oscillation (NAO) is the major atmospheric mode that controls winter European climate variability because its strength and phase determine regional temperature, precipitation and storm tracks. The NAO spatial structure and associated climatic impacts over Europe are not stationary making it crucial to understanding its past evolution in order to improve the predictability of future scenarios. In this regard, there has been a dramatic increase in the number of studies aimed at reconstructing past NAO variability, but the information related to decadal-scale NAO evolution beyond the last millennium is scarce and inconclusive. We present a new 2,000-year multi-annual, proxy-based reconstruction of local NAO impact, with associated uncertainties, obtained by a Bayesian approach. This new local NAO reconstruction is obtained from a mountain lacustrine sedimentary archive of the Iberian Peninsula. This geographical area is not included in previous NAO reconstructions despite being a widely used region for instrumental-based NAO measurements. We assess the main external forcings (i.e., volcanic eruptions and solar activity) on NAO variability which, on a decadal scale, show that a low number of sunspots correlate to low NAO values. By comparison with other previously published NAO reconstructions in our analyses we can test the stationarity of the solar influence on the NAO signal across a latitudinal gradient based on the position of the employed archives for each NAO reconstruction. Inconclusive results on the volcanic forcing on NAO variability over decadal time-scales indicates the need for further studies. Moreover, we highlight the potential role of other North Atlantic modes of variability (i.e., East Atlantic pattern) on the non-stationary behaviour of the NAO throughout the Common Era, likely via solar forcing.

## Introduction

The North Atlantic Oscillation (NAO) is characterised by a dipole of sea-level pressure (SLP) anomalies between the Azores and Iceland^1^ ^and references therein^. Under positive NAO conditions, when the dipole is enhanced, storm tracks shift towards N Europe bringing more precipitation and warm anomalies into this region. Conversely, negative NAO conditions induce average temperature and more precipitation in S Europe. The NAO drives regional climates on different spatio-temporal scales, and controls important socio-economic activities. Climate change imposes new societal challenges and adaption strategies, so understanding NAO variability is key to wind-energy production, food security and important ecosystem services like global terrestrial CO_2_ uptake and water availability^2–4^. However, the NAO impact is non-stationary on decadal timescales^5^ making its reconstruction a challenge for proxy-based records beyond the instrumental period. Recent dynamical method developments offer promise to improve seasonal NAO forecasting^6^ but assessing its predictability on decadal timescales requires documentation of past low-frequency NAO variability.

**Figure 1:**
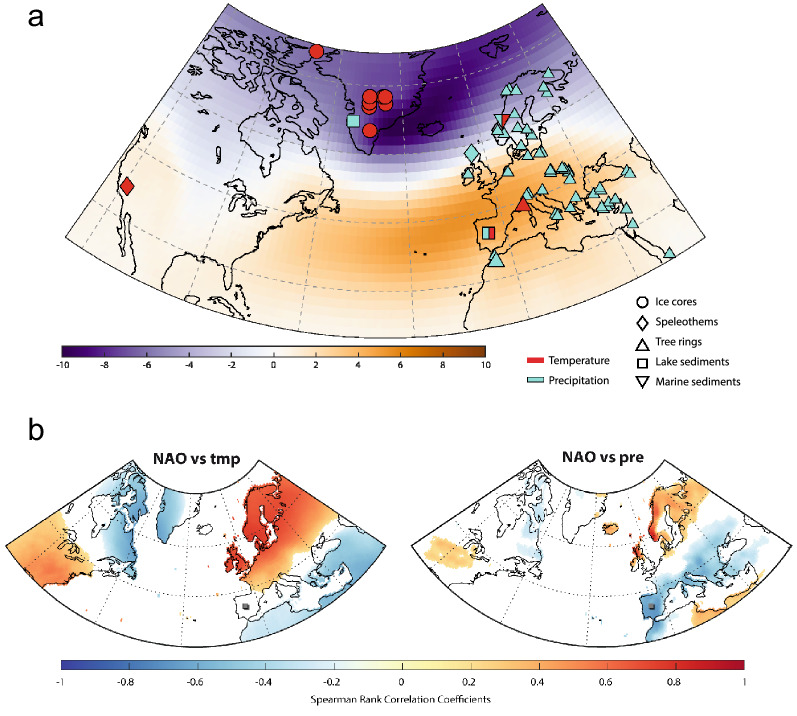
(**a**) Spatial display of the first eigenvector for the gridded winter (December–February) monthly SLP anomalies (in mb) for the North Atlantic domain—calculated using the Twentieth Century Reanalysis data set (20CRv2c)^77^. Location of the proxy-based records (ice cores, lake sediments, speleothems, tree rings, and marine sediments) employed in this study, using symbols and colours to represent the different types of archives and the reconstructed climate variables. (**b**) Correlation distribution maps between the winter precipitation and temperature (wPre and wTmp) datasets and the NAO, for the boreal winters (December–February) between 1901 and 2016, calculated using the CRU-TS4.1 global climate dataset^69^ and the NAO and EA indices from Comas-Bru and Hernández^57^. Positive Spearman rank correlations are shown in red and negative correlations are shown in blue. Location of record used for NAO_IP_ is indicated. Figure created with MATLAB 2019b. Scripts at https://doi.org/10.5281/zenodo.3898382.

This importance of the NAO for explaining European climate variability (Fig. 1) has encouraged a number of initiatives to produce NAO reconstructions across different timescales^7–14^. However, these are still challenged within the paleoclimate community, partly because there is a widespread use of ambiguous terminology. Encouraging the use of more accurate definitions (i.e., local impacts vs regional reconstructions) would help to understand the discrepancies amongst NAO reconstructions. For instance, while a reconstruction that assembles a large dataset of multi-archive sub-decadal proxy records from different locations over the region of influence of the NAO (e.g., Greenland, Mediterranean, Scandinavia) could provide stronger constraints^13,15,16^ on NAO variability (Fig. 1), each archive (e.g., ice cores, tree-rings, speleothems) may be recording a different aspect of the NAO according to their seasonal sensitivity, for example. Additionally, the extent to which the NAO impacts the climate may differ across locations, further reducing the ability to obtain a robust NAO reconstruction from a geographically spread multi-archive dataset. In fact, many studies use just one or two records^11,12,17,18^ (Fig. 1) to obtain a more accurate (avoiding over-smoothing) yet spatially limited reconstruction of the local NAO impact. Thus, the use of more accurate definitions according to the employed methodology would facilitate the determination of some of the current discrepancies between NAO reconstructions.

In general, most of the available proxy-based NAO reconstructions (Table 1) agree with each other on centennial timescales, and demonstrate broad agreement with instrumental NAO indices until as early as c. 1850 CE (Common Era). However, NAO variability only accounts for c. 40% of the climate variance that is ultimately captured by those regional palaeorecords sensitive to the NAO impact^1^. Thus, on this basis a perfect NAO reconstruction is a challenge beyond the instrumental period. In fact, considerable discrepancies amongst reconstructions are evident further back in time on decadal timescales. A clear example is the persistent positive NAO phases during the Medieval Climate Anomaly (MCA; 900–1300 CE) suggested by some authors^11,12,17^ and questioned by others^13,16^. Besides factors such as chronological uncertainties, the use of diverse calibration periods and differences in the sensitivity of the archives to climate, amongst others; the inconsistencies between NAO reconstructions appear to be related to the large percentage of NAO internal variability that has been commonly associated with its non-stationary behaviour^19,20^. More recently, the amplitude of this internal variability has been attributed to the influence of other North Atlantic modes of climate variability [i.e., the East Atlantic (EA) and Scandinavian (SCA) patterns], which would thereby modulate the strength and location of the NAO dipole from annual to multidecadal scales^21–23^. The EA pattern is structurally similar to the NAO, being defined as a north–south dipole with SLP anomaly centres, spanning the entire North Atlantic Ocean^3,24^, or a well-defined SLP monopole south of Iceland and west of Ireland^4,22,23,25^. Compared with the nodal lines of the NAO pattern, the anomaly centres of the EA pattern are displaced to the southeast^26^. The SCA pattern is associated with strong positive SLP anomalies over Scandinavia and weaker centres of the opposite sign over Western Europe and eastern Russia–western Mongolia^27^.

**Table 1:** Previous NAO index reconstructions used in this work.

References	Reconstruction Period	Time Resolution	Predictors	Statistical Method
Luterbacher et al.^9^ (NAO_LUT_)	1659–1995 CE 1500–1658 CE	Monthly Seasonal	Instrumental and proxy data predictors from Eurasia	Principal Component Regression (PCR)
Trouet et al.^11^ (NAO_TRO_)	c. 1050–2000 CE	Seasonal	Reconstructed winter precipitation for Scotland and February-to-June Palmer Drought Severity Index (PDSI) for Morocco	Normalised difference of the Scotland and Morocco records
Olsen et al.^12^ (NAO_OLS_)	c. 3250 BCE–1650 CE	Decadal	Paleo-redox proxy-based record of lake sediments from southwestern Greenland	3^rd^ component of a Principal Component Analysis (PCA) tuned by Monte-Carlo-Markov-Chain model (calibration based on Trouet et al. 2009)
Ortega et al.^13^ (NAO_ORT_)	c. 1050–2000 CE	Annual	Proxy data predictors (ice cores, lake sediment, speleothem and tree ring) from around the North Atlantic (Greenland, Europe, North America and North Africa)	Ensemble reconstruction of Principal Component Regressions (PCR)
Baker et al.^17^ (NAO_BAK_)	1000 BCE–2000 CE	Annual	Speleothem	Principal Component Analysis (PCA)
Faust et al.^18^ (NAO_FAU_)	c. 800 BCE–1900 CE	Multiannual	Paleoproductivity (CaCO3 and Ca/Si) proxy-based record of fjord sediments from Central Norway	Kernel smoother
Sjolte et al.^15^ (NAO_SJO_)	c. 1250–2000 CE	Seasonal	Reconstruction of atmospheric winter circulation for the North Atlantic region based on Greenland ice core records and a 1,200-year-long simulation with an isotope-enabled climate model	Principal Component Analysis (PCA) supported by a Chi-square goodness-of-fit test
Cook et al.^16^ (NAO_COOK_)	910–2018 CE	Seasonal	Tree rings	Principal components regression

From a methodological point of view, most previously published NAO reconstructions have been based on the use of some variant of regression models, often coupled with Principal Components Analysis (PCA)^14^ ^and references therein^. By contrast, richer models using Bayesian inference have been extensively used during the last decade for age-depth chronological building^28–30^ as well as for climate and environmental reconstructions using biological proxies^31–34^. Nevertheless, neither Bayesian inference nor non-biological proxies have yet been used to reconstruct modes of variability (i.e., the NAO). The Bayesian approach holds a major advantage over traditional methods, as it is conceptually simpler to build a complex model which quantifies the relationship between multiple proxy and climate variables simultaneously—rather than relying on individual coefficients to describe the relationship^35,36^. Furthermore, it is possible to model observations under all conditions (i.e., modern analogues^37^). The handicap for ''no modern analogue'' situations means considerably larger uncertainties which can be, however, accounted for in the resulting reconstructions.

Recently, much attention has been cast toward disentangling the relative controls on the NAO from external forcings (e.g., solar, volcanic activity and/or greenhouse gases) and internal variability (e.g., ocean, atmosphere, sea ice) to develop reliable projections of its future evolution^6,38–42^. Although the relative impacts of external forcing mechanisms on the NAO are still a matter of debate^43^, it has been traditionally assigned to volcanic eruptions^44^, as highlighted by a predominance of positive NAO phases after these periods of increased volcanic activity^13,15^. The role of solar activity however is even more controversial, with contradictory evidence sourced from multiple proxy-based reconstructions^13,15,18,45^. Modelling^42,46^ and observational^40,47^ studies also yield contradictory conclusions between the 11-year solar cycle and the NAO relationship^48^. The studies arguing for a solar impact on the NAO invoke a top-down mechanism related to the ultraviolet irradiance pattern^49^. An increase in UV radiation during periods of high solar activity results in an increased temperature in the middle atmosphere. The middle atmosphere refers to the region extending from the tropopause (∼ 10–16 km) to the homopause (∼ 110 km) where the atmosphere remains relatively well mixed^50^. This increase in UV radiation due to high solar activity would lead to an altered stratospheric circulation that propagates pole- and down-wards affecting tropospheric jet streams and thus atmospheric circulation^40,51^. However, the response of the NAO to the solar cycle would not occur immediately but rather after a lag of c. 3 years. This is because the impact of solar heating accumulates for several years in the ocean causing a positive feedback between the ocean and atmosphere^49,52^. Low sunspot activity results in a climate pattern very similar to the negative phase of the NAO^53^ with longer lasting and more intense blocking episodes than during high solar blocking events^54^. A recent proxy-based study^18^ further supports a linkage between the Grand minima of solar activity and the negative NAO phases that accompany cooling events (e.g., Little Ice Age—LIA) at decadal-to-centennial timescales.

Here we present a quantitative NAO reconstruction for the central Iberian Peninsula (IP) over the last two millennia, along with its uncertainties, by applying a Bayesian approach. We also assess the coherence between our new local NAO reconstruction and previously published reconstructions from other locations, as well as potential external forcing mechanisms that would lead to disagreements between them as a result of the non-stationary spatial behaviour of the NAO.

## A new local NAO reconstruction: Central Iberian Peninsula

The NAO has a significant effect on winter climate on the Iberian Peninsula^55–57^ (IP; Fig. 1). In particular, high-mountain lakes from the IP are highly influenced by the NAO; cold and wet conditions during negative NAO phases control annual ice-cover dynamics (i.e., freezing and thawing) via interactions between air temperature and precipitation^58^. While the NAO is particularly relevant during the boreal winter, its impact on ecosystem and ice-cover dynamics is not restricted to this season^59^. In fact, the NAO signal that is captured in lake records from the region spans from January to May^58^.

A previous study^60^ using geochemical (i.e., X-ray fluorescence and bulk organic matter nitrogen (TN), carbon (TC) and analyses of their stable isotopes analyses), as well as mineralogical (i.e., x-ray diffraction) data from the Cimera Lake sedimentary record (40° 15′ N–5° 18′ W, 2,140 m a.s.l.) established a qualitative climatic and environmental reconstruction of the Iberian Central Range throughout the CE. Authors applied PCA to the normalized geochemical datasets to determine the main environmental processes controlling sediment input, distribution and deposition in the lake. The first (PC1cim) and second (PC2cim) eigenvectors explained c. 55% of the total variance. PC1cim was associated with most of the chemical elements and with Ti in particular. Therefore, it was interpreted as due to changes in the inputs of siliciclastic material from the catchment. Authors argued that runoff intensity was related to the occurrence of a well-defined melt season in terms of temperature and rainfall variations. Hence, rain-on-snow events were suggested as the main process governing the inputs of coarse siliciclastic material to Cimera Lake. PC2cim was related to TC, TN and Rb content and was associated with variations in the lake’s organic productivity which was in turn modulated by ice-cover duration. Cold (warm) and wet (dry) conditions lead to longer (shorter) ice-cover durations, which are partly the result of the enhanced (reduced) insulating effect of the snow deposited on the ice cover^58^. Here we use their dataset^60^ to quantitatively reconstruct the NAO impact on climate in the central IP (NAO_IP_) for the last two millennia using a Bayesian modelling approach (see Methods).

**Figure 2:**
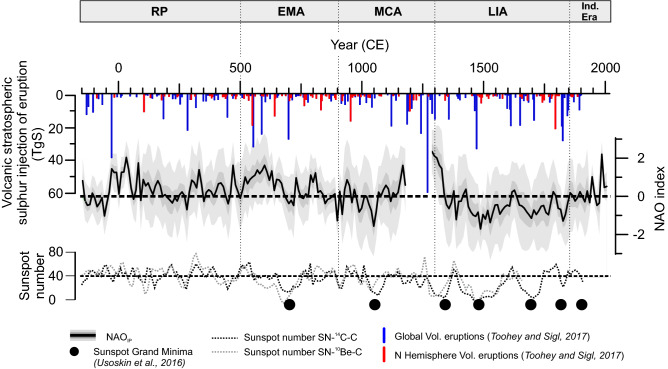
The NAO reconstruction for the Central Iberian Peninsula (NAO_IP_) obtained in this study (black line) and the 95% (light grey band) and 50% (dark grey band) uncertainty intervals. Sunspot number reconstruction^62^ and volcanic eruption^61^ for the studied period are also represented.

**Figure 3:**
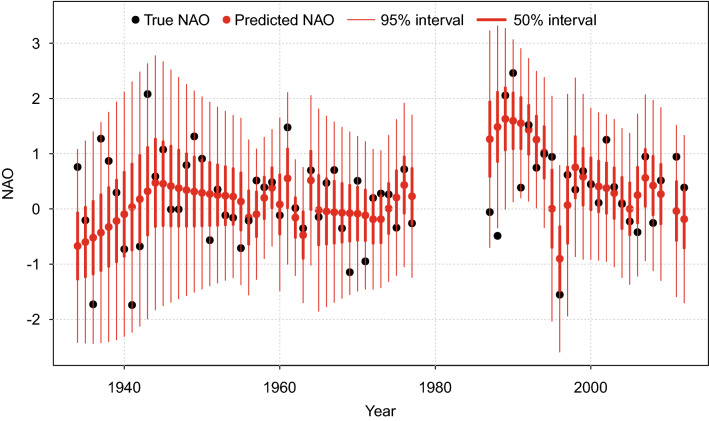
Black points correspond to instrumental NAO data (i.e., NAO index data) and the red points show predicted median NAO impact values. The vertical red lines correspond to the 95% uncertainty interval (thin lines) and to the 50% uncertainty interval (wide lines), respectively.

The reconstructed local NAO impact ranges between − 3 and 3 and represents the quantitative reconstruction of the NAO for the central IP (NAO_IP_; Fig. 2). Only 2.9% of the observations fall outside the 95% confidence interval (Fig. 3). These results indicate satisfactory performance of the model and validate the NAO_IP_ (see Methods). The NAO_IP_ has not been reconstructed for the period c. 1200–1270 CE due to the lack of proxy data^60^. Though the model permits such interpolation, the uncertainties would be too wide to enable any reasonable interpretation.

The NAO_IP_ shows decadal alternations between positive (> 0.5) and neutral (− 0.5 to 0.5) phases of the NAO during the Roman Period (RP: ~ 200 BCE–500 CE) (Fig. 2). Neutral to positive NAO_IP_ values characterize the first half of the RP period while there is a predominance of neutral values during its second half. During the Early Middle Ages (EMA: 500–900 CE) the NAO_IP_ shows two cycles of positive-to-neutral and positive-to-negative (< − 0.5) values. During the Medieval Climate Anomaly (MCA: 900–1300 CE), the NAO_IP_ displays a trend from predominantly negative values (− 1.4 to 0.5) to the most positive ones (~ 2.5) of the entire reconstruction. Negative NAO_IP_ values (− 1.6 to 0.5) clearly dominate the Little Ice Age (LIA: 1300–1850 CE) recording the most negative NAO_IP_ values at ~ 1500 CE. In contrast, the Industrial Era (IE: 1850–2012 CE) shows a clear trend from neutral to positive values (− 0.2 to 0.6) punctuated by large decadal oscillations (~ 2.5) during the second half.

## Comparison with previous NAO proxy-based reconstructions

**Figure 4:**
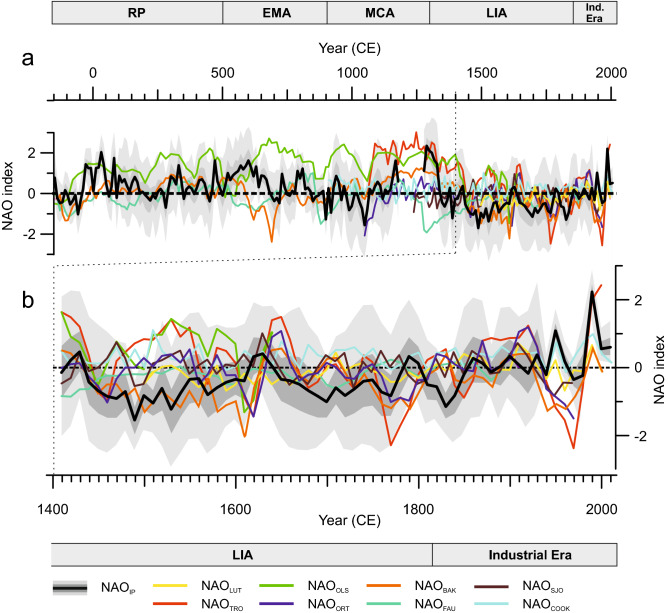
(**a**) Comparison of the NAO reconstructions for decadal timescales. Details of each reconstruction are in Table 1. (**b**) Magnified plot for the last six centuries.

The comparison between different proxy-based NAO reconstructions published in the last two decades (Table 1) points out a number of periods with consistent signals as well as some with notable differences (Fig. 4). All NAO reconstructions show a similar centennial timescale evolution with positive NAO values during the MCA and lower positive or negative NAO values during the LIA (Fig. 4). This coherence across regions suggests an hemispheric imprint of this climate mode at low frequencies compared to the local impacts that might be recorded by each reconstruction over annual to decadal timescales.

To establish the extent to which the decadal variability between positive and negative excursions in NAO_IP_ during the last 2 ka compares to previously published reconstructions, we calculated Spearman's Rank Correlation Coefficients for decadal timescales (Table 2; Fig. 4). The NAO_IP_ displays the highest correlations with the W Europe mid-latitude records such as the NAO reconstruction by Trouet et al.^11^ (NAO_TRO_; ρ = 0.51; *p* < 0.01; DF = 171) and the NAO reconstruction by Baker et al.^17^ (NAO_BAK_; ρ = 0.40; *p* < 0.01; DF = 206)_._ However, if we analyse the results separately for climate periods, the NAO_IP_ shows higher correlations with high-latitude and eastern records such as NAO reconstruction by Ortega et al.^13^ and NAO reconstruction by Faust et al.^18^ during the MCA (ρ = 0.84; *p* < 0.01; DF = 25) and the IE (ρ = 0.94; *p* < 0.01; DF = 15), respectively.

**Table 2:** Spearman's rank correlation coefficients between decadal (10 years) NAO_IP_ and other NAO proxy-based reconstructions employed to build up NAO indices.

		NAO_**LUT**_	NAO_**TRO**_	NAO_**OLS**_	NAO_**ORT**_	NAO_**BAK**_	NAO_**FAU**_	NAO_**SJO**_	NAO_**COOK**_
NAO_**IP**_	CE (200 BCE—Present)	0.18^d^	**0.51**^**a**^	0.20^b^	0.22^b^	**0.40**^**a**^	0.08^d^	0.13^d^	0.07^d^
	RP (200 BCE–500 CE)	–	–	0.24^d^	–	0.30^b^	0.19^d^	–	–
	EMA (500–900 CE)	–	–	− 0.31^c^	–	0.18^d^	− 0.19^d^	–	–
	MCA (900–1300 CE)	–	0.28^d^	0.30^d^	**0.84**^**a**^	0.57^a^	− 0.05^d^	0.50^d^	− 0.26^d^
	LIA (1300–1850 CE)	− 0.22^d^	0.29^b^	0.27^d^	0.06^d^	0.35^a^	− 0.33^b^	0.13^d^	0.10^d^
	IE (1850 CE—Present)	0.36^d^	0.11^d^	–	0.17^d^	0.27^d^	**0.94**^**a**^	0.01^d^	0.28^d^

Correlations with *p* val < 0.01 are in bold.

^a,b,c,d^Correlations with *p* val < 0.01, 0.01 < *p* val < 0.05, 0.05 < *p* val < 0 .1 and *p* val > 0.1, respectively.

**Figure 5:**
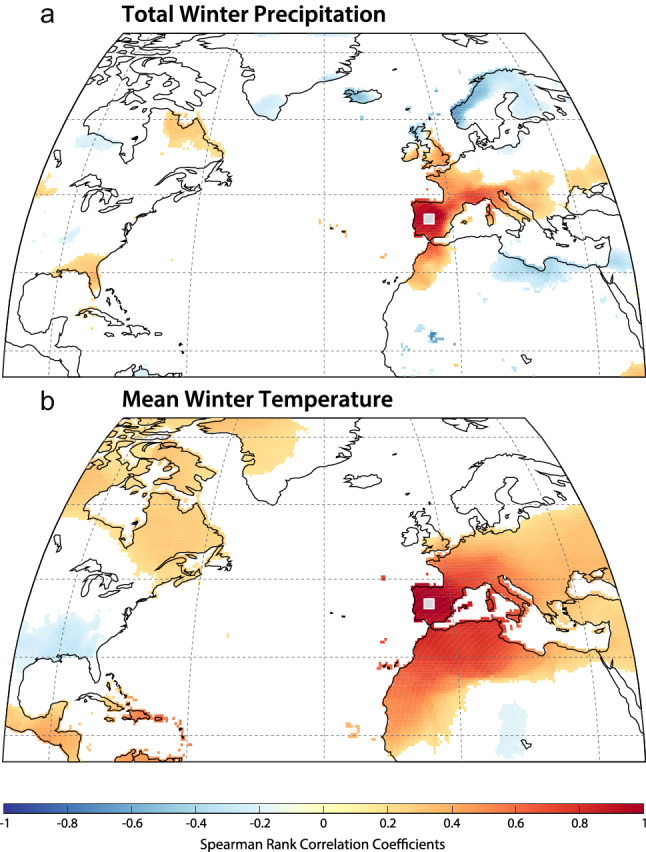
Correlation between winter (**a**) precipitation and (**b**) temperature at the site of NAO_IP_ (grey square) and each of the other grid cells to see how representative is the site for regional winter climate between 1901 and 2016, calculated using the CRU-TS4.1 global climate dataset^69^. Positive Spearman rank correlations are shown in red and negative correlations are shown in blue. Figure created with MATLAB 2019b. Scripts at https://doi.org/10.5281/zenodo.3898382.

Unlike instrumental-based NAO reconstructions arising from large-scale gridded SLP datasets proxy-based NAO reconstructions are usually based on a limited number of climate proxy records from restricted regions or locations (e.g., NAO_TRO_, NAO_BAK_, NAO by Sjolte et al.^15^ (NAO_SJO_)) or on single paleorecords (e.g., NAO reconstruction by Olsen et al.^12^, NAO_FAU_ and our NAO_IP_). This constrained geographical area implies that the reconstructed NAO signal probably results from the local climatic response to this mode of variability rather than reflecting a regional signal. By contrast, other NAO reconstructions, such as those by Cook et al.^16^ (NAO_COOK_), Luterbacher et al.^9^ (NAO_LUT_) and NAO_ORT_ are based on a larger number of geographically distributed records that could be understood to represent a regional assemblage of local NAO impacts. Nevertheless, here we provide evidence supporting the fact that our local NAO_IP,_ which is based on a single palaeorecord, is representative of a wide regional signal (Fig. 5). Thus, we conclude that in some cases, reconstructions based on single archives may be more regionally representative than what can be achieved with multi-archive proxy-based NAO reconstructions. The rationale behind this could be that different archives capture different climate signals at different seasons and merging them into a single series without taking into account these mixed signals results in a low variance reconstruction that does not capture all the NAO variability.

**Figure 6:**
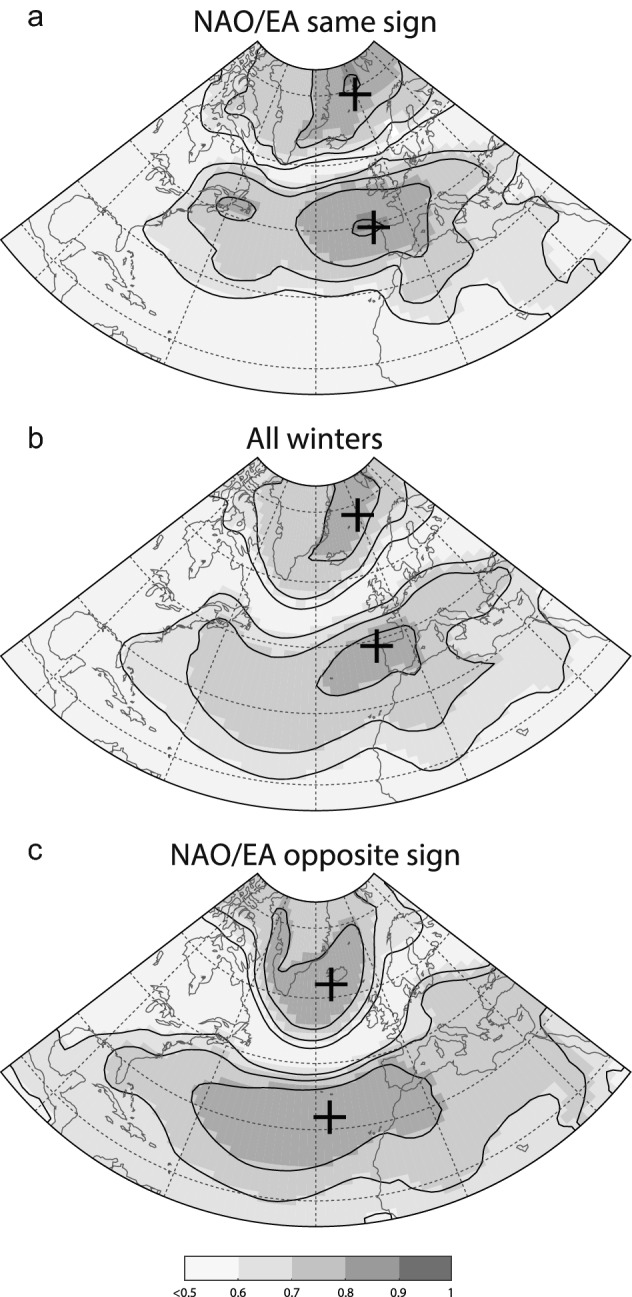
Teleconnectivity maps of the winter (DJF) monthly SLP field in the North Atlantic region for the period 1872–2009 and different linear combinations of the NAO-EA: (**a**) winters with the NAO and the EA of the same sign; (**b**) all winters; (**c**) winters with the NAO and the EA of the opposite sign. Shaded areas represent Spearman correlations as per the colour bar. Black crosses indicate the location of the highest correlated grid cells. The NAO and the EA indices are the 1st and 2nd empirical orthogonal functions calculated from monthly SLPanomalies over a confined N. Atlantic sector using the Twentieth Century Reanalysis data set (20CRv2c)^77^. Figure created with MATLAB 2019b. Scripts at https://doi.org/10.5281/zenodo.3898382.

**Figure 7:**
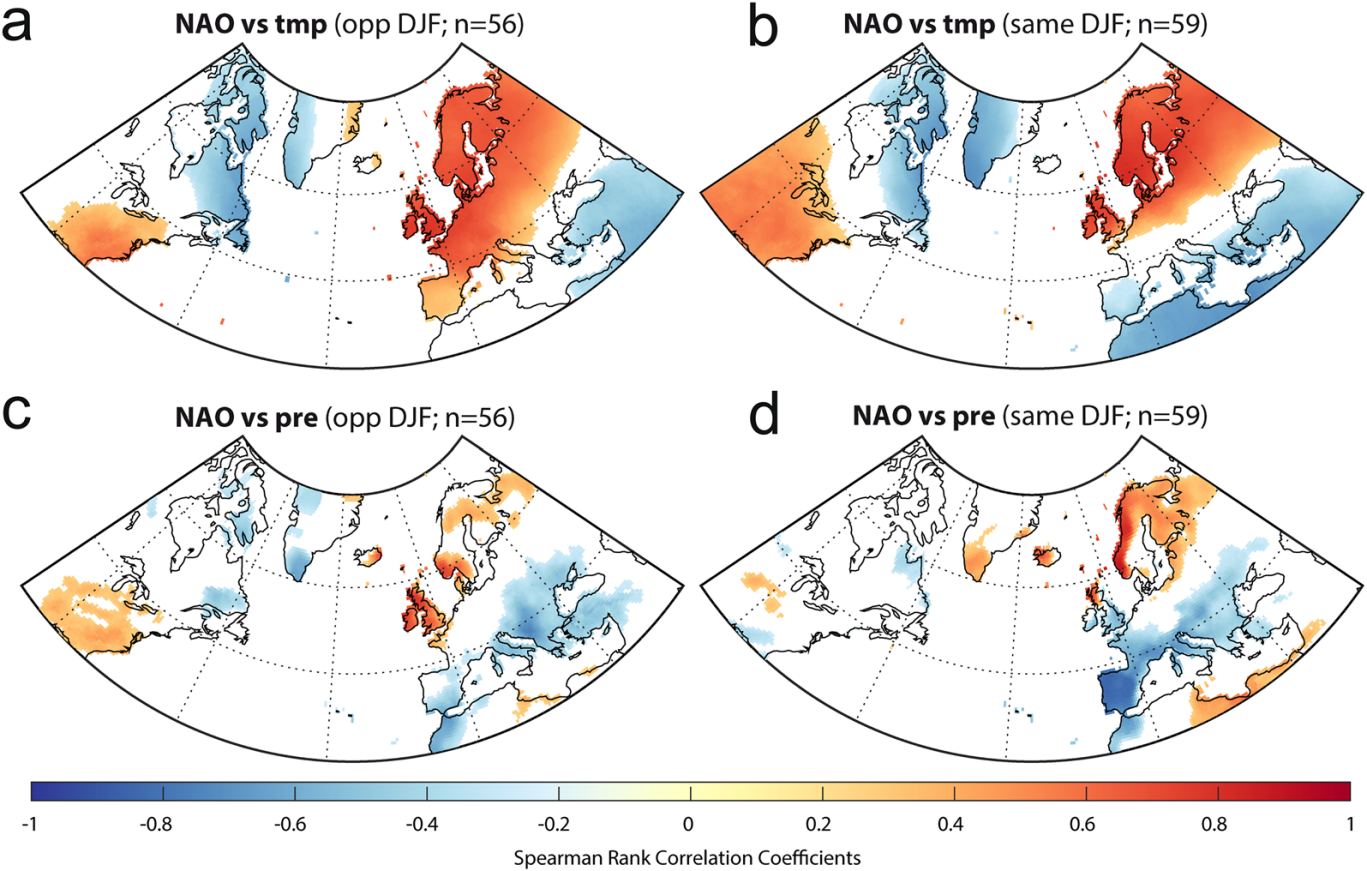
Correlation distribution maps of winter NAO and (**a**, **b**) temperature and (**c**, **d**) precipitation. (**a**, **c**) are correlations for the subset of winters where NAO and EA are of opposite sign, whereas (**b**, **d**) are correlations for the subset of winters where NAO and EA are of the same sign. NAO and EA indices from Comas-Bru and Hernandez^57^ and climate data from the CRU-TS4.01 dataset^69^. Location of NAO_IP_ is shown in Figs. 1 and 5. Figure created with MATLAB 2019b. Scripts at https://doi.org/10.5281/zenodo.3898382.

Equally importantly, the NAO is not the sole driver of the large-scale atmospheric variability in the European North-Atlantic region; other modes of variability also play an important role—namely the EA and SCA^56^. Comas-Bru and McDermott^23^ previously showed that it is possible to explain a larger fraction of the European winter climate variability when different NAO/EA and NAO/SCA modulate the migrations of the North Atlantic SLP dipole and in turn, the climate signal recorded in the region. Across the IP, this is expressed by a more homogeneous spatial pattern in temperature and precipitation for periods with a predominance of in-phase NAO and EA indices (i.e., MCA and LIA) compared to periods when these modes have the opposite sign (i.e., RP and EMA)^60^. Moreover, the geographical displacement of the southern pole of the North Atlantic SLP field (i.e., location of the highest correlated grid cells between combinations of the NAO and EA and North Atlantic SLP) is relatively smaller when the NAO/EA have the same sign compared to years of opposite sign (Fig. 6). Contour lines of teleconectivity maps show higher gradients and are northerly located for years with the same sign, whereas years with opposite sign show a lower gradient with a south-westerly migration of the dipole. As a result, differences in precipitation between years of the NAO and the EA with the same and opposite signs (Fig. 7) are generally small for mid-latitudes (e.g., IP and the Mediterranean Basin) and are larger for high-latitudes (e.g., Greenland, Ireland, and the UK). On the contrary, differences in temperature are larger for mid-latitudes and smaller for high-latitudes. These differences are almost non-existent in the western Atlantic sector (US and Canada) where the NAO impact is also significantly weaker. Hence, regional NAO reconstructions which use different archives (e.g., tree-rings, speleothems, ice-cores) that record different climate variables (e.g., precipitation, temperature) from different locations (high- vs mid-latitudes) could experience biases due to the above mentioned migrations of the SLP dipole due to NAO/EA interactions. This could potentially explain the latitudinal variability observed for NAO reconstructions over the last millennia (Fig. 4).

The largest discrepancies between NAO_IP_ and other published reconstructions are found for the RP and the EMA, while better agreements occur during the MCA, the LIA, and the IE (Table 2, Fig. 4). During the RP and the EMA, the weakest agreement occurs for NAO reconstructions that are based on high latitude records (NAO_OLS_ and NAO_FAU_) whereas a similar long-term evolution is observed between NAO_IP_ and NAO_BAK_, which are based on mid-latitude records (Figs. 1, 4). While we acknowledge the difficulty to fully assess the reasons for these discrepancies, our results indicate that they may be partly related to the location of the employed archives (higher latitudes for NAO_OLS_ and NAO_FAU_ than for NAO_IP_ and NAO_BAK_); potentially it is also due to the different latitudinal impact of the EA pattern, as well as external forcings. Nonetheless, the type of archives employed for the different reconstructions (i.e., lake and marine sediments, speleothems), their variable sensitivity to climate, and the range of statistical approaches used to obtain the final reconstructions (i.e., Bayesian modelling, Principal Component Analysis), should not be discarded as being at least part of the cause of the disagreements observed across reconstructions.

## Volcanic eruption impact

Previously published proxy-based NAO reconstructions show a robust positive NAO response in the 4–5 years following the major eruptions of the last millennial period^13,15^. In contrast, a recent review of the impact of explosive volcanic eruptions on the main climate variability modes determined that no firm conclusions can be drawn regarding volcanic forcing impacts on this mode of variability^43^.

We compared the NAO_IP_ with volcanic eruptions^61^ responsible for the largest stratospheric sulphur injection (> 6 Tg S) during the last 2 ka for each decade (n = 41; Table 3). We find positive reconstructed NAO values for approximately 50% of the decades when these large eruptions occurred. This result reinforces previous studies^13,15^ that did not reach compelling conclusions on the relationship between the NAO and volcanic activity. Nevertheless, if we relax the minimum injection threshold for volcanic eruptions in order to take into account stratospheric sulphur injections larger than 0.5 Tg S and only consider Northern Hemisphere latitudes eruptions (n = 86), we find that about 80% of the NAO reconstructed values of the decades encompassing these eruptions are predominantly positive (c. 80%). A further comparison including all the analysed NAO reconstructions (Table 1) shows a wide range of percentages (20 to 91% and 32 to 91% for all and for extratropical NH eruptions, respectively) for decades with positive NAO values when large volcanic eruptions occurred (Table 3). Hence, there is no apparent influence from volcanic eruptions on the preferred signal of the NAO pattern over decadal timescales.

**Table 3:** Percentage of positive NAO decades after the largest global (volcanic stratospheric sulphur injection > 6 TgS) and NH (volcanic stratospheric sulphur injection > 0.5 TgS) volcanic eruptions following Toohey and Sigl^61^.

	Global (150 BCE–2012 CE)	NH (150 BCE –2012 CE)
**NAO**_**IP**_	**18/36 (50%)**	**48/60 (80%)**
NAO_LUT_	2/10 (20%)	7/22 (32%)
NAO_TRO_	15/202 (75%)	21/34 (64%)
NAO_OLS_	20/22 (91%)	51/56 (91%)
NAO_ORT_	10/20 (50%)	14/34 (41%)
NAO_BAK_	23/40 (58%)	46/87 (53%)
NAO_FAU_	13/40(33%)	37/87 (43%)
NAO_SJO_	8/16 (50%)	15/26 (58%)
NAO_COOK_	20/24 (83%)	36/43 (84%)

NAO_IP_ is in bold.

## Solar forcing modulation

We also compared the NAO_IP_ with a decadal sunspot number reconstruction^62^ through the CE. To avoid volcanic eruption interferences in the analysis, we identified the decades corresponding to the fifteen largest volcanic eruptions (Fig. 2 and Table S1), considered to be outliers, and were removed from the solar forcing analysis.

**Figure 8:**
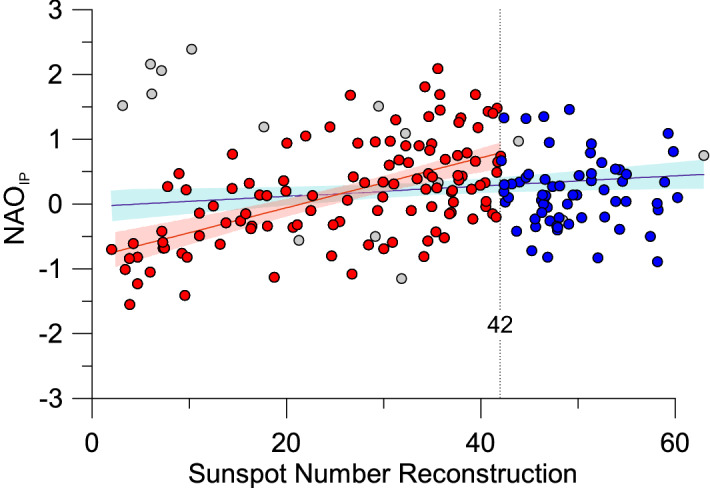
NAO_IP_ values versus sunspot number reconstruction. Red dots represent samples with sunspot number values below 42, whereas blue dots indicate samples with sunspot number values above 42. Grey dots correspond to samples during decades with high volcanic eruptions and they have not been considered in the correlation analysis. Red line indicates linear correlation between NAO_IP_ and Sunspot number (< 42) and blue line for all data. Dotted line delimits samples with sunspot number values below and above 42.

The linear correlation (ρ) between the decadal NAO_IP_ index and the sunspot number is significant but relatively low, (ρ = 0.32; *p* < 0.01; DF = 177). However, a breakpoint analysis displayed an inflection point with the occurrence of 42 sunspots (Fig. 8). If we only consider sunspot numbers under 42, the linear correlation with NAO_IP_ is also significant and much higher (ρ = 0.57; *p* < 0.01; DF = 112). This correlation is higher in the last millennium (1000–2012 CE; ρ = 0.59; *p* < 0.01; DF = 48), where the NAO_IP_ shows negative values between (1020–1070 CE; 1450–1550 CE; 1640–1720 CE and 1810–1850 CE) corresponding to Oort, Spörer, Maunder, and Dalton Grand solar minima, respectively, than in the previous millennium (0–1000 CE; ρ = 0.28; *p* > 0.1; DF = 60) with almost no Grand solar minima (Fig. 2).

To evaluate this relationship, we reproduced the same analysis for all the NAO reconstructions (Table 4 and Fig. S1). The results are similar to those obtained for the NAO_IP_ with low or non-significant correlation values when using all sunspot numbers (Table 4). However, those proxy-based NAO reconstructions where mid-latitude records have a prevailing role (e.g., NAO_BAK_ and NAO_TRO_) also displayed a tipping point at a sunspot number of 42 (Fig. S1). In these cases, there is also a significant correlation between the decadal NAO values and this sunspot number (ρ > 0.22; *p* < 0.01; DF > 50). To test whether the NAO behaviour is indeed significantly influenced by solar activity for sunspot numbers below 42 for reconstructions based on lower latitude records, we have also compared the southernmost record of the NAO_TRO_—Palmer Drought Severity Index (PDSI) based on tree ring data from Morocco^63^—with the sunspot number. This comparison confirms a significant correlation (ρ = 0.34; *p* < 0.01; DF = 51) between solar activity and the NAO for mid-latitudes. These latitudinal differences in solar activity impact can be attributed, among other criteria previously mentioned (e.g., type of proxy and methodology), to the fact that the solar variability signal is not uniformly distributed^64^. Annual and decadal variations in solar activity have the largest impacts in the mid-latitudes^65^. Previous analyses of surface air temperatures^66^ have demonstrated a tendency toward preferential warming in regions at 30°–60° latitude for both hemispheres.

**Table 4:** Spearman’s rank correlation coefficients from the preformed linear regression models between proxy-based NAO reconstructions and Sunspot Number reconstruction. Decades with large volcanic eruptions have been removed from the analysis. In bold, significant correlation values at 0.01 significance. Note that NAO_TRO_ from Morocco (in italics) is included to highlight the solar activity impact in lower latitudes.

	SSN (150 BCE–2012 CE)	SSN < 42 (150 BCE–2012 CE)
**NAO**_**IP**_	**0.32**	**0.57**
NAO_LUT_	0.03	0.10
NAO_TRO_	**0.14**	**0.29**
*NAO*_*TRO (Morocco)*_	*0.11*	***0.33***
NAO_OLS_	**0.10**	0.01
NAO_ORT_	0.15	0.03
NAO_BAK_	0.09	**0.22**
NAO_FAU_	**0.19**	**0.23**
NAO_SJO_	0.22	0.07
NAO_COOK_	0.08	0.04

## Discussion and conclusions

There is an evident historical disagreement between all the available proxy-based reconstructions over decadal timescales for the Common Era. While there is a clear consensus for prevailing negative NAO phase conditions during the Little Ice Age and positive conditions for the Medieval Climate Anomaly over centennial timescales; discrepancies emerge when NAO variability is analysed at annual-to-decadal timescales. Moreover, the scarcity of NAO reconstructions at decadal resolutions makes it difficult to identify common patterns before the last millennium. Our results suggest mainly positive and neutral phases of the NAO during the Roman Period (~ 200 BCE–500 CE), and two cycles: i) positive-to-neutral; and ii) positive-to-negative values during the Early Middle Ages (500–900 CE).

New applied statistical approaches (i.e., Bayesian) will help to improve the reliability of these results. We also suggest a more adaptable concept of proxy-based NAO reconstruction using appropriate distinctions, since it is impossible to understand the NAO as a single pattern. Rather, it should be regarded as a complex system that is controlled by multiple factors, some of which are stochastic and are therefore difficult to constrain. For this, the interpretation of available NAO reconstructions requires a careful re-examination. The sensitivity of different climate archives to the NAO may vary over different spatio-temporal scales. This highlights the need to select archives which are more sensitive to winter climate parameters (larger NAO impact season) and localities where the NAO impact on local climate variables is more stable (stationary).

Beyond these methodological issues, we have also analysed the distinct impact of potential external forcings (i.e., volcanic eruptions and solar activity) to puzzle out the reasons underpinning the observed disagreements between the NAO reconstructions throughout time.

Our results demonstrate that solar activity influences NAO variability over decadal timescales. The NAO reconstructions based on proxy records from mid-latitudes display significant positive correlations with the sunspot number, but this relationship is only found up to a certain solar activity threshold (number of sunspots < 42), after which the NAO index appears to be less influenced by solar activity. On the contrary, the impact of volcanic eruptions on the NAO is less clear, with disparate percentages showing some dominant positive NAO values after large volcanic eruptions.

Besides the influence of these two external forcing mechanisms we also assessed the role of an internal mechanism—namely the interaction of the NAO with the second most important large-scale pattern of atmospheric circulation in the North-Atlantic European sector, i.e., the Eastern Atlantic (EA) pattern. Combinations of NAO and EA phases can change the geographical position of the NAO centres of action and affect the strength and latitudinal location of the dominant westerlies entering Europe from the Atlantic^21^. Thus, the sensitivity of the archive to record the NAO impact (i.e., seasonality, climate variable, resolution) and its location are crucial to more accurately reconstruct NAO variability. Although a wide regional distribution of records could probably yield better results, the contrary impact of combined NAO and EA modes on some climate variables for the mid- and high-latitude records could be masking or, even, cancelling out the actual NAO pattern.

Further studies are required to better understand the NAO’s behaviour and the disagreements between the continuously increasing number of available NAO reconstructions. Regional NAO reconstructions like the ones derived by integrating a grid of instrumental or proxy-based regional data can be considered more robust—and can aid the understanding of general climate dynamics—only if the records employed are sensitive to the same forcing and therefore can capture the same signal. In contrast, local NAO reconstructions would be more useful for determining its impacts on local meteorological variables, being more relevant for local ecosystems and the socio-economic system. Therefore, local NAO reconstructions can help to develop better mitigation policies against problems derived from NAO climatic effects such as agricultural yield or water scarcity.

## Data and methods

### Proxy data

We used the chemical composition of a lacustrine sediment core (CIM12-04A, 124.8 cm long) retrieved from an alpine lake located in the Central Iberian Range (Cimera Lake, 40° 15′ N–5° 18′ W, 2,140 m a.s.l.)^60^. The chemical composition of the sediments was obtained by continuous X-ray fluorescence (XRF) analysis using the XRF Avaatech core scanner located at the University of Barcelona (Spain). The XRF settings (working conditions) were: 2 mm of spatial resolution, 2 mA, 15 s count times and 10 kV for lighter elements, with 55 s and 30 kV for heavier elements. Thirty chemical elements were measured, but only ten light (Al, Si, K, Ca, Ti, V, Cr, Mn, Fe, and Zn) and three heavy (Rb, Sr and Zr) elements had enough counts to be considered robust.

The chronology of the sediment deposition of the CIM12-04A core was previously determined by Sánchez-López et al.^60^. It was derived using the activity-depth profile of ^210^Pb in the uppermost 9 cm of the core together with six AMS ^14^C dating. The resulting model shows that sedimentary infill of the Cimera Lake core spans from 172 ± 65 BCE to 2012 CE. See details in Supplementary Material.

### Climate datasets

We used the NAO extended winter index (Jan–May) spanning the period 1824–2012 CE to produce the NAO influence reconstruction. The data were obtained from the Climatic Research Unit (CRU) at the University of East Anglia (UK) (https://crudata.uea.ac.uk/cru/data/pci.htm). This NAO index was defined by Jones et al.^67^ and modified by Vinther et al.^68^ to be the difference between the normalized monthly SLP anomalies recorded at Reykjavik (Iceland) and those observed at Iberia (Gibraltar/Cádiz). Precipitation and temperature datasets used in the figures were obtained from CRU-TS4.01^69^, whereas NAO and EA data were acquired from Comas-Bru and Hernández^57^.

### Bayesian model

We follow a Bayesian modelling approach^31,70^ to produce a reconstruction of the NAO’s impact on the central IP. The relationship between proxy and climate is derived from a training data set for the instrumental/proxy calibration period and is expressed through a likelihood function. This function is combined with a prior probability density function containing parameter information in order to obtain a posterior probability distribution of the reconstructed NAO values using Bayes’ theorem^71^. Whilst Parnell et al.^31^ based their framework on reconstructing multivariate temperature and moisture measurements from raw pollen data, the method is easily adaptable to other proxies and climate variables. Indeed, Cahill et al.^34^ used a similar approach to reconstruct sea level from foraminifera. In all cases the measurements/counts of the proxy are required for a set of sediment layers (depths) in a core.

We summarize the mathematical details of the model in this section. Full technical details for the model fitting process are described in Parnell et al.^31^. We provide all the code used to create the reconstructions at www.github.com/andrewcparnell/NAO.

The notation we use is as follows:

- *NAO(t)* is the North Atlantic Oscillation value at time *t*. The goal of our model is to estimate *NAO(t)*, and its uncertainty, for a set of chosen times.
- *XRF*_*ij*_ (expressed as counts per second) represents the chemical element *j* measured at a given depth *i* of the CIM12-04A core. We have *i* = 1,…, 647 depths and *j* = 1,…, 13 elements.
- We superscript both the quantities above with *m* and *f* so that *XRF*^*m*^ refers to the modern XRF data set, with associated known *NAO*^*m*^, and *XRF*^*f*^ refers to the fossil data set, for which we wish to estimate *NAO*^*f*^.
- *t*_*i*_ refers to the age of the core at depth *i*. The ages in our core are all given in years BC/AD.
- *θ* refers to the set of parameters governing the relationship between the *NAO* and the *XRF* measurements, as well as the dynamics of how the *NAO* changes over time.

Our model proceeds by creating a Bayesian joint posterior distribution:

$$ {p\left( {NAO^{f} ,\theta |NAO^{m} ,XRF^{m} ,XRF^{f} } \right) \propto p\left( {XRF^{f} |NAO^{f} } \right) \cdot p\left( {XRF^{m} |NAO^{m} ,\theta } \right) \cdot p\left( {NAO^{f} |\theta } \right) \cdot p\left( \theta \right)} $$

The term on the left-hand side of the equation is the posterior distribution and represents the probability distribution of fossil NAO impacts given the observed data. The terms on the right-hand side represent respectively, the likelihood (the probability distribution of *XRF*^*f*^ given *NAO*^*f*^), the distribution of *XRF*^*m*^ given *NAO*^*m*^, and the prior distribution of the parameters governing the relationship between *XRF* and *NAO.*

For the distribution of *XRF* given the *NAO*, we standardise all the *XRF* values (by chemical element) and fit a multivariate normal polynomial regression model (MVN). This means, for the values *k* = *1,…, 13* chemical elements, we use:

$${\left[ {XRF_{i,1} ,\ldots,XRF_{i,13} } \right]|NAO\left( {t_{i} } \right) \sim MVN\left( {M_{i} ,\Sigma } \right)}$$

where *Mi* = *[μ*_*i,1,…,*_*μ*_*i,13*_*]* with *μ*_*ik*_ = *β*_*0k*_ + *β*_*1k*_* NAO(t*_*i*_*)* + *β*_*2k*_* NAO(t*_*i*_*)*^2^, and ∑ is a covariance matrix which captures the extra dependence between elements not explained by differences in the NAO.

We set the prior distribution on *NAO*^*f*^ as a continuous-time random walk, which should reasonably match climate behaviour over the reconstructed time period (as in Haslett et al.^32^). Other choices are available, such as long-memory or long-tailed stochastic processes^31^ use a Normal inverse Gaussian process. Our prior distribution is:

$${NAO\left( {t_{i} } \right)\sim N\left( {NAO\left( {t_{{i - {1}}} } \right),\sigma^{2} \left( {t_{i} - t_{{i - {1}}} } \right)} \right)}$$

where σ^2^ is a parameter representing the variance of the *NAO*^*f*^ increments for a unit of time.

Finally, we set uninformative prior distributions for the remaining parameters:

$${\beta_{{{0}k}} , \beta_{1k} , \beta_{2k} \sim N\left( {0,10} \right), \sigma \sim U\left( {0,10} \right), \Sigma^{ - 1} \sim Wishart\left( {I_{{{13}}} {, 14}} \right)}$$

where *N* and *U* represent Normal and Uniform distributions and *I* is the identity matrix.

The above model is computationally expensive to fit using the default tools for Bayesian model fitting due to the large number of parameters and the high data dimension. Instead, as stated above, we follow the approach of Parnell et al.^31^, which uses a computational approximation to fit the model in three steps. The first step fits the model to the modern data only. The second step estimates *NAO*^*f*^ for the fossil layers, and the third step constrains the estimated fossil *NAO*^*f*^ values according to a random walk model.

In the first step of the model, the total overlapping period between modern data, XRF proxy and observed NAO index (i.e., *NAO*^*m*^ and *XRF*^*m*^, respectively), extends from 1825 until 2012 AD. However, the XRF data from 1825 until 1930 CE has a lower resolution (i.e., decadal) than the 1930–2012 CE period, owing to the usual slight decrease in the age-depth model accuracy. Thus we restricted the overlapping fitting period employed in the analysis to 1930–2012 CE. *XRF*^*m*^ data were resampled with a yearly resolution for the overlapping period using the R function "approxTime" from the package “simecol”^72^.

We fit the model in R^73^ using the JAGS software^74^ (Just Another Gibbs Sampler). The performance of the fitting algorithm can be determined by looking at the Brooks-Gelman Rubin $(\hat{\text{R}})$ statistic^75,76^ as well as trace plots of the parameter samples for each iteration of the algorithm. We run the algorithm until all $\hat{\text{R}}$ values are less than 1.05, which indicates satisfactory convergence of the algorithm to the posterior distribution.

We evaluate the performance of the model by testing the predictive performance of the modern relationship between *NAO*^*m*^ and *XRF*^*m*^ (Step 1 as outlined above). As validation procedure, we compared NAO predicted values from the model using the modern NAO data (i.e., NAO instrumental data; Fig. 3). If the model estimates the NAO correctly there should be only 5% of the observations outside the 95% interval, and 50% outside the 50% interval. Finally, the complete impact reconstruction is created using a 10-year time grid, and includes both 95% and 50% uncertainty intervals.

### Statistical analyses

All NAO reconstructions have been converted to decadal time-scales to facilitate comparison. For each reconstruction, all NAO values within the same decade have been averaged and we use that average value for its particular decade. The magnitude of the relationships between NAOs were obtained according to Spearman’s rank correlation coefficients (ρ) and associated p values. Unless otherwise stated, significance (*p* value) is always considered at values of *p* < 0.01.

We have analysed the NAO values for each decade with significant volcanic eruptions for the last 2 k years according to two different thresholds^61^: (1) eruptions around the globe with more than 6 Tg S injected to the troposphere, and (2) eruptions from the Northern Hemisphere with accumulations larger than 0.5 Tg S injected to the troposphere. We have established a percentage of positive NAO values that occurred over the decades selected for each threshold.

Relationships between solar forcing and the NAO were established with linear regression models and verified with a number of diagnostic techniques (see supplementary material). We also applied two thresholds: (1) all the sunspot number reconstruction values and, (2) values lower than 42. The latter was selected after we applied a breakpoint analysis which delivered the sunspot number of 42 as a potential breakpoint in the regression line (see supplementary material).

The statistical treatment of the data was performed with R software^73^.

## Supplementary information

Supplementary Information.

## Data Availability

The proxy-based NAO dataset along with its uncertainties is available at https://doi.pangaea.de/10.1594/PANGAEA.921916.
