# Supplementary material for: A 2,000-year Bayesian NAO reconstruction from the Iberian Peninsula
Source: Sci Rep. 2020 Sep 11;10:14961. doi: 10.1038/s41598-020-71372-5 (PMC7486925; doi:10.1038/s41598-020-71372-5)
Supplement: Supplementary file 1 — Supplementary Information. [file 41598_2020_71372_MOESM1_ESM.pdf]

Supplementary information

A 2,000-year Bayesian NAO reconstruction from the Iberian Peninsula

Armand Hernandez[1+*], Guiomar Sánchez-López[1+], Sergi Pla-Rabes[2], Laia Comas-Bru[3], Andrew Parnell[4], Niamh Cahill[5], Adelina Geyer[1], Ricardo M Trigo[6,7], Santiago Giralt[1].

[1]Geosciences Barcelona (GEO3BCN-CSIC), Barcelona, Spain

[2]CREAF, Campus de Bellaterra (UAB), Edifici C, 08193, Cerdanyola del Vallès

[3]School of Archaeology, Geography and Environmental Sciences, University of Reading, Reading, UK

[4]Hamilton Institute, Insight Centre for Data Analytics, Maynooth University, Kildare, Ireland

[5]Department of Mathematics and Statistics, Maynooth University, Maynooth, Kildare, Ireland.

[6]Instituto Dom Luiz (IDL), Faculdade de Ciências, Universidade de Lisboa, 1749-016, Lisboa, Portugal

[7]Departamento de Meteorologia, Universidade Federal do Rio de Janeiro, 21941-916, Rio de Janeiro, Brasil

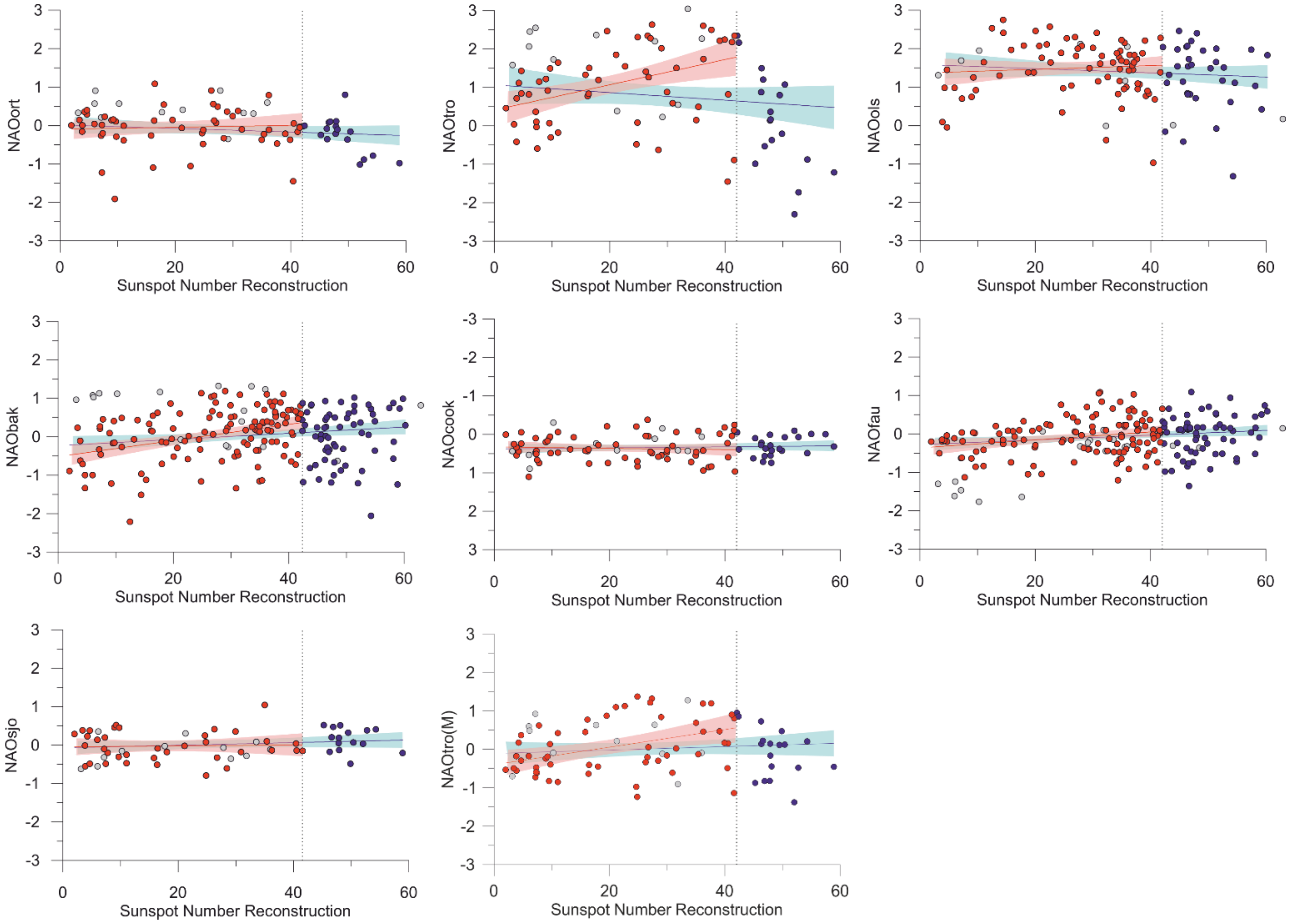

Fig. S1: NAO values versus sunspot number reconstruction. Red dots represent samples with sunspot number values below 42, whereas blue dots indicate samples with sunspot number values above 42. Grey dots correspond to samples during decades with high volcanic eruptions; they have not been considered in the correlation analysis. Red line indicates linear correlation between NAO and Sunspot number (< 42) and blue line for all data.

Table S1: Fifteen global largest volcanic eruptions (according to Sigl et al.[1]) removed from the solar activity analysis

Year (CE)	Eruption
1815	Tambora (Indonesia)
1809	UE 1809
1783	Laki (Iceland)
1601	Huaynaputina (Peru)
1458	Kuwae (Vanuatu)
1258	Samalas (Indonesia)
1230	UE 1230
1108	UE 1108
682	Pago? (New Britain)
574	Rabaul? (New Britain)
540	Ilopango? (El Salvador)
536	UE 536
426	UE-426
266	UE 266
44 BCE	Chiltepe? (Nicaragua)

Chronology

The chronological framework of CIM12-04A was derived using the 210Pb activity-depth profile together with AMS 14C dating pollen concentrates. The concentration profile of 210Pb in the uppermost 20 cm of the core was determined by quantifying its decay product 210Po by alpha-spectroscopy following Sánchez-Cabeza et al.[2]. The supported 210Pb concentration was estimated by averaging the concentration of 210Pb below 9 cm, where it remained constant because there was not enough material available to conduct measurements by gamma spectrometry. $210Pb_{ex}$-derived sediment accumulation rates were calculated by applying the constant flux: constant sedimentation model (CF:CS)[3]. The pollen concentrates were obtained following Rull et al.[4]. Radiocarbon ages were calibrated to calendar years (BP and CE/BCE) using the online CALIB 7.1 software[5] and INTCAL13 curve[6] by selecting the median of the 95.4% distribution (2s probability interval) (Table S1). Finally, the age-depth relationship for the CIM12-04A model was established using the R-code package 'clam' and a smooth spline (type 4) with a 0.3 smoothing value and 1000 iterations[7]. The concentration profile of the excess 210Pb can be divided into three intervals according to its slopes: 0-4 cm, 4-6.5 cm and 6.5-9 cm. The supported 210Pb horizon is attained at 9.2 cm. Relatively lower concentrations of $210Pb_{ex}$ were measured in the sandy layers and excluded for the estimation of the sedimentation rates for each zone (Fig. S2). The sedimentation rates (SRs) were obtained by applying the CF:CS model. The concentration of 210Pbex was constant in the 6.5-8.7 cm interval; thus, a chronology could not be derived. Seven AMS 14C dates were determined between 10 and 124.5 cm (Table S2). The date at 10 cm was too old for its stratigraphic location, which was most likely a result of the reworking of the older sediment. Therefore, this sample was not used in the construction of the age-depth model (Fig. S3). Four distinct SR intervals can be differentiated according to the age depth model: a) 4.1 mm/yr for 0-4 cm, b) 0.65 mm/yr for 4-6.5 cm, c) 0.2 mm/yr for 6.5-29.5 cm and d) 0.9 mm/yr for 29.5-124.5 cm. The SRs from the two first intervals were calculated by applying the CF:CS model, whereas the third and fourth intervals were obtained by linear interpolations between dates. The resulting age-depth model shows that the uppermost 124.5 cm of sedimentary infill spans from ca. 200 BCE to 2012 CE (Fig. S3).

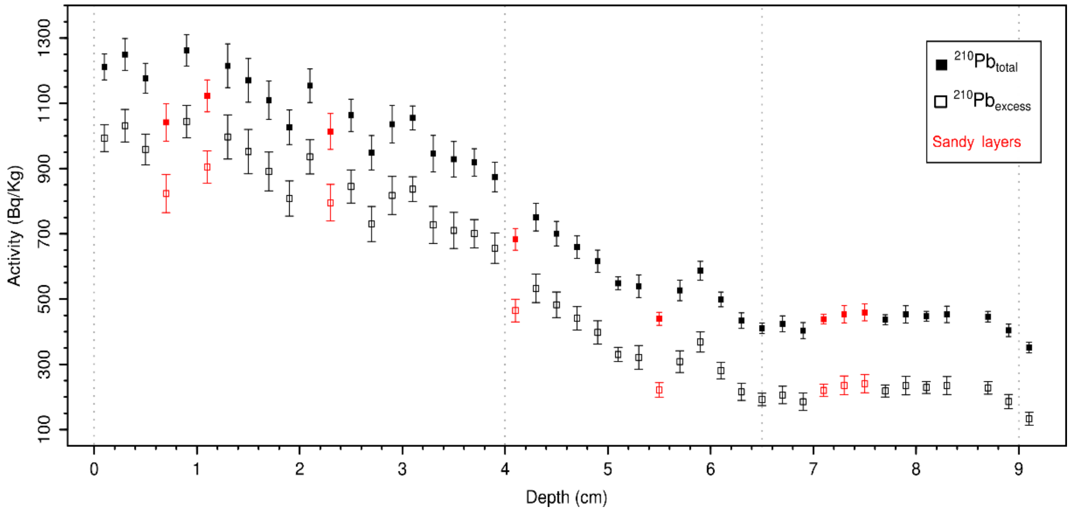

Fig. S2. Concentration profiles of the total and excess 210Pb for the CIM12-04A core[8]; error bars represent 1s uncertainties. The vertical dashed lines delimit the three intervals of the concentration profile of the excess 210Pb according to its slopes.

Table S2: Radiocarbon dates and calibrated ages for the CIM12-04A core[8]. Results are reported with 2s uncertainty. The sample in grey (shaded) and italics was discarded.

Depth (cm)	Lab Code	Material	**^{14}C years BP**	Cal. years BP (2σ)	Cal. years AD/BC (2σ)
10	Beta - 333752	Pollen concentrate	*1420 ± 30*	*1329 ± 39*	*621 ± 39 AD*
29.5	Poz-61896	Pollen concentrate	1140 ± 30	1032 ± 63	918 ± 63 AD
49	Beta - 333753	Pollen concentrate	1170± 30	1113 ± 66	837 ± 66 AD
67.5	Poz-61897	Pollen concentrate	1425 ± 30	1332 ± 40	618 ± 40 AD
86	Beta - 333754	Pollen concentrate	1940 ± 30	1885 ± 64.5	64.5 ± 64.5 AD
105.5	Poz-61898	Pollen concentrate	1875 ± 30	1803.5 ± 76.5	146.5 ± 76.5 AD
124.5	Beta - 333755	Pollen concentrate	2160 ± 30	2122.5 ± 65.5	172.5 ± 65.5 BC

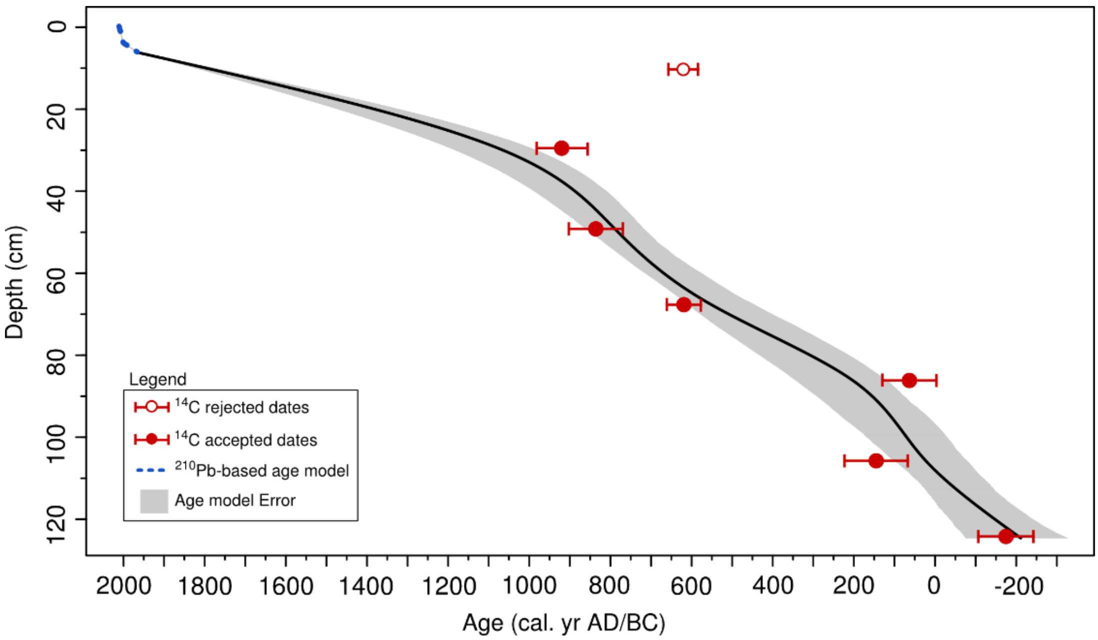

Fig. S3. Age-depth model based on the AMS 14C dates and 210Pb activity-depth profile[8]. Error bars for the red points represent the ±2 sigma calibrated age range for AMS 14C dates. The black continuous line represents the age-depth function framed by the grey area, which corresponds to the error model. The blue dashed line represents the zone of 210Pb dates.

Linear regression models and validation tests to establish the NAO and sunspot number reconstruction relationship

<u>NAOip SSN (150 BCE - 2012 CE)</u>

```
Call:
lm(formula = NAOip ~ SSN...0, data = NAOip_SSN0)

Residuals:
     Min       1Q   Median       3Q      Max 
-1.41805 -0.44476 -0.06661  0.39345  1.89908 

Coefficients:
             Estimate Std. Error t value Pr(>|t|)    
(Intercept) -0.339694   0.124426  -2.730  0.00697 ** 
SSN...0      0.014917   0.003295   4.527  1.1e-05 ***
---
Signif. codes:  0 ‘***’ 0.001 ‘**’ 0.01 ‘*’ 0.05 ‘.’ 0.1 ‘ ’ 1

Residual standard error: 0.6529 on 177 degrees of freedom
Multiple R-squared:  0.1038,	Adjusted R-squared:  0.0987 
F-statistic: 20.49 on 1 and 177 DF,  p-value: 1.096e-05


Anova Table (Type II tests)

Response: NAOip
          Sum Sq  Df F value    Pr(>F)    
SSN...0    8.735   1  20.492 1.096e-05 ***
Residuals 75.451 177                      
---
Signif. codes:  0 ‘***’ 0.001 ‘**’ 0.01 ‘*’ 0.05 ‘.’ 0.1 ‘ ’ 1
```

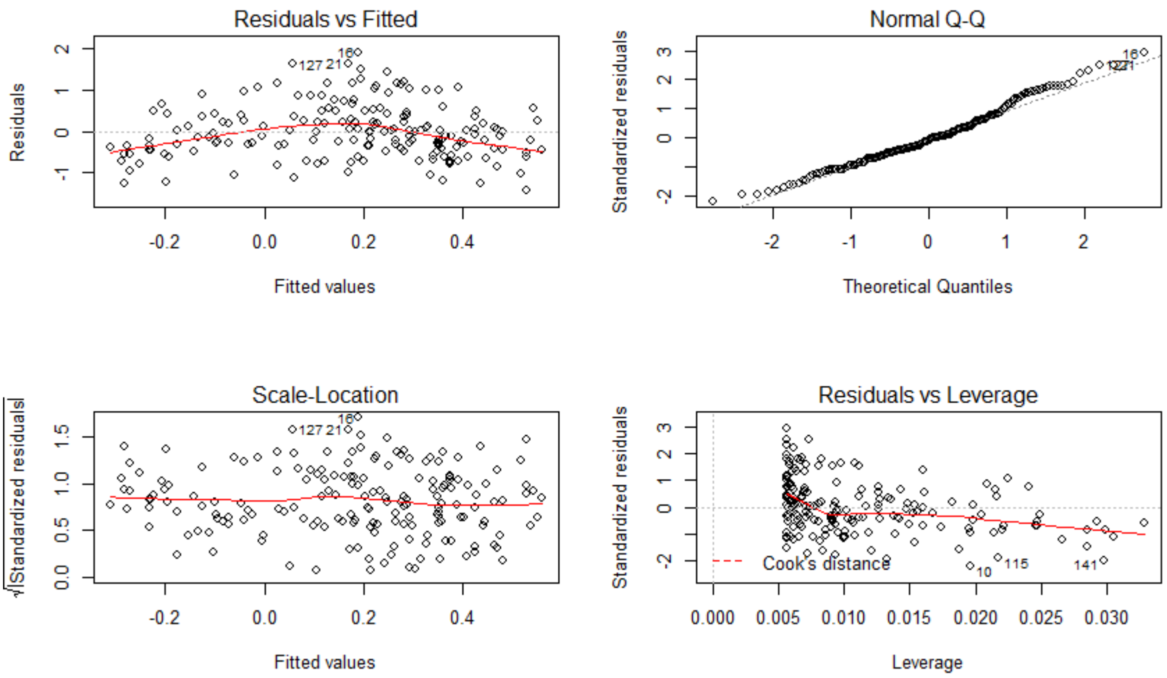


```
Multiple regression power calculation

              u = 1
              v = 178
             f2 = 0.1160714
      sig.level = 0.01
          power = 0.974546
```

NAOip SSN<42 (150 BCE - 2012 CE)

```
Call:
lm(formula = NAOip ~ SSN, data = NAOip_SSN42)

Residuals:
     Min       1Q   Median       3Q      Max 
-1.26704 -0.38175 -0.06615  0.44083  1.58098 

Coefficients:
             Estimate Std. Error t value Pr(>|t|)    
(Intercept) -0.793078   0.145245  -5.460 2.89e-07 ***
SSN          0.036607   0.005016   7.298 4.50e-11 ***
---
Signif. codes:  0 ‘***’ 0.001 ‘**’ 0.01 ‘*’ 0.05 ‘.’ 0.1 ‘ ’ 1

Residual standard error: 0.634 on 112 degrees of freedom
Multiple R-squared:  0.3223,	Adjusted R-squared:  0.3162 
F-statistic: 53.26 on 1 and 112 DF,  p-value: 4.499e-11


Anova Table (Type II tests)

Response: NAOip
          Sum Sq  Df F value    Pr(>F)    
SSN       21.408   1  53.262 4.499e-11 ***
Residuals 45.018 112                      
---
Signif. codes:  0 ‘***’ 0.001 ‘**’ 0.01 ‘*’ 0.05 ‘.’ 0.1 ‘ ’ 1
```

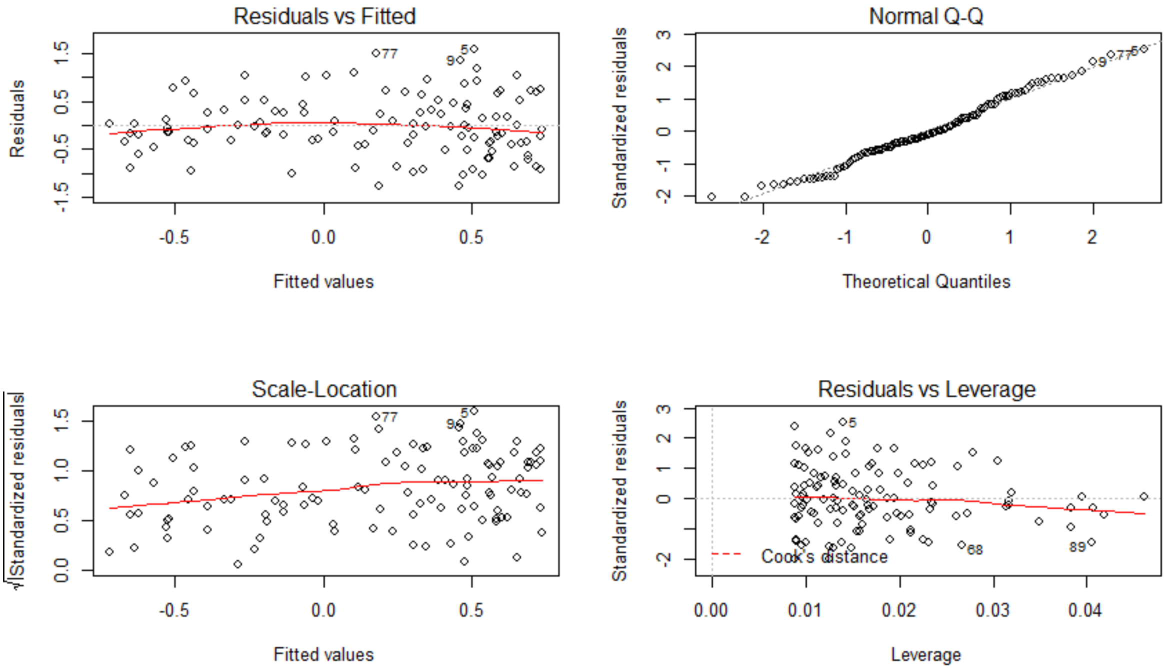


```
Multiple regression power calculation 
              u = 1
              v = 113
             f2 = 0.4755792
      sig.level = 0.01
          power = 0.9999988
```

NAOip SSN<42 (1400 - 2012 CE)

```
Call:
lm(formula = NAOip ~ SSN, data = NAOip_SSN42_600)

Residuals:
     Min       1Q   Median       3Q      Max 
-0.82903 -0.28466  0.01015  0.23500  1.08019 

Coefficients:
             Estimate Std. Error t value Pr(>|t|)    
(Intercept) -0.805972   0.121646  -6.626 2.46e-07 ***
SSN          0.021851   0.005799   3.768 0.000719 ***
---
Signif. codes:  0 ‘***’ 0.001 ‘**’ 0.01 ‘*’ 0.05 ‘.’ 0.1 ‘ ’ 1

Residual standard error: 0.4099 on 30 degrees of freedom
Multiple R-squared:  0.3212,	Adjusted R-squared:  0.2986 
F-statistic:  14.2 on 1 and 30 DF,  p-value: 0.0007194


Anova Table (Type II tests)

Response: NAOip
          Sum Sq Df F value    Pr(>F)    
SSN       2.3857  1  14.198 0.0007194 ***
Residuals 5.0410 30                      
---
Signif. codes:  0 ‘***’ 0.001 ‘**’ 0.01 ‘*’ 0.05 ‘.’ 0.1 ‘ ’ 1
```

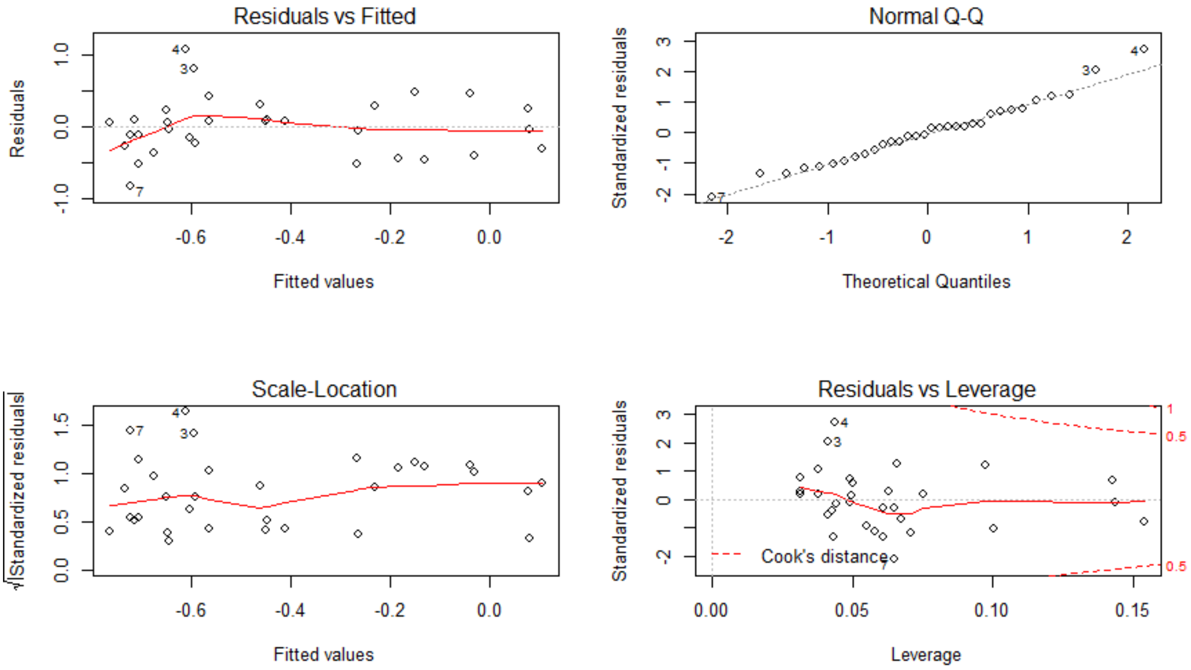


```
 Multiple regression power calculation 

              u = 1
              v = 31
             f2 = 0.473188
      sig.level = 0.01
          power = 0.8772529
```

NAOlut SSN (150 BCE - 2012 CE)

```
Call:
lm(formula = Luter ~ SSN...0, data = NAOlut_SSN0)

Residuals:
     Min       1Q   Median       3Q      Max 
-0.52724 -0.20181  0.01905  0.16648  0.61506 

Coefficients:
              Estimate Std. Error t value Pr(>|t|)  
(Intercept) -0.1682456  0.0860035  -1.956   0.0582 .
SSN...0      0.0004373  0.0024750   0.177   0.8607  
---
Signif. codes:  0 ‘***’ 0.001 ‘**’ 0.01 ‘*’ 0.05 ‘.’ 0.1 ‘ ’ 1

Residual standard error: 0.2756 on 36 degrees of freedom
  (179 observations deleted due to missingness)
Multiple R-squared:  0.0008665,	Adjusted R-squared:  -0.02689 
F-statistic: 0.03122 on 1 and 36 DF,  p-value: 0.8607


Anova Table (Type II tests)

Response: Luter
           Sum Sq Df F value Pr(>F)
SSN...0   0.00237  1  0.0312 0.8607
Residuals 2.73398 36
```

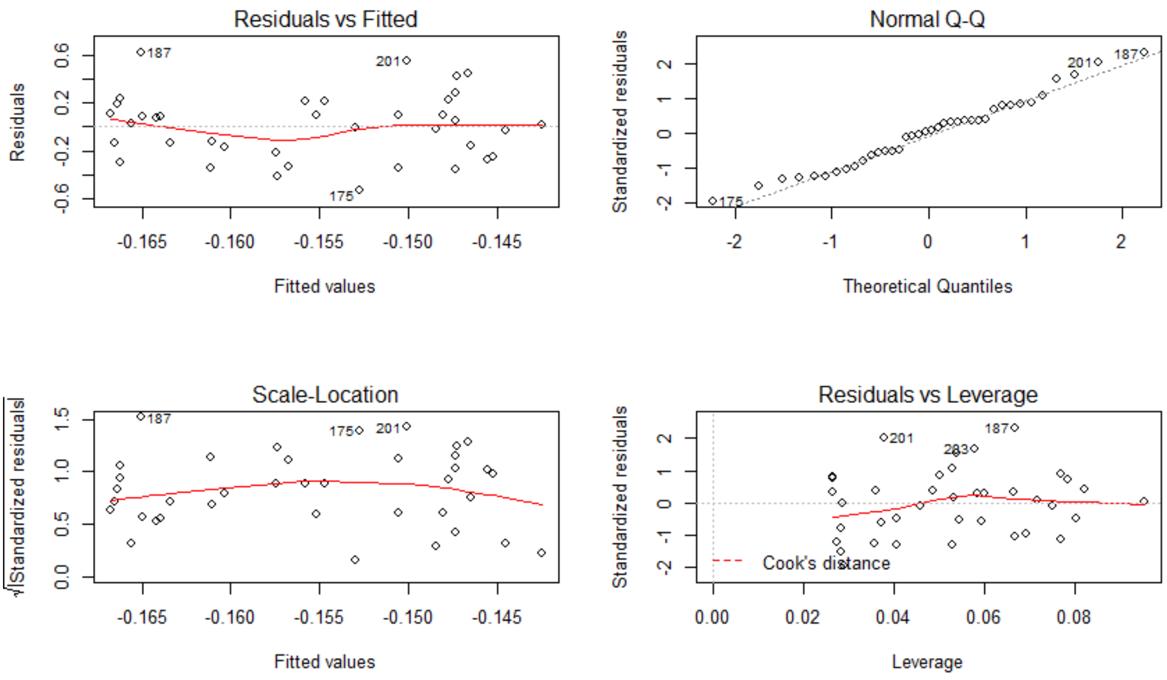


```
 Multiple regression power calculation 

              u = 1
              v = 37
             f2 = 0.001001001
      sig.level = 0.01
          power = 0.01134416
```

NAOlut SSN<42 (150 BCE - 2012 CE)

```
Call:
lm(formula = Luter ~ SSN, data = NAOlut_SSN42)

Residuals:
     Min       1Q   Median       3Q      Max 
-0.46768 -0.18051  0.05149  0.16623  0.62548 

Coefficients:
             Estimate Std. Error t value Pr(>|t|)
(Intercept) -0.137217   0.102302  -1.341    0.193
SSN         -0.002122   0.004408  -0.481    0.635

Residual standard error: 0.2866 on 23 degrees of freedom
  (110 observations deleted due to missingness)
Multiple R-squared:  0.00997,  Adjusted R-squared:  -0.03307 
F-statistic: 0.2316 on 1 and 23 DF,  p-value: 0.6349


Anova Table (Type II tests)

Response: Luter
           Sum Sq Df F value Pr(>F)
SSN       0.01902  1  0.2316 0.6349
Residuals 1.88878 23
```

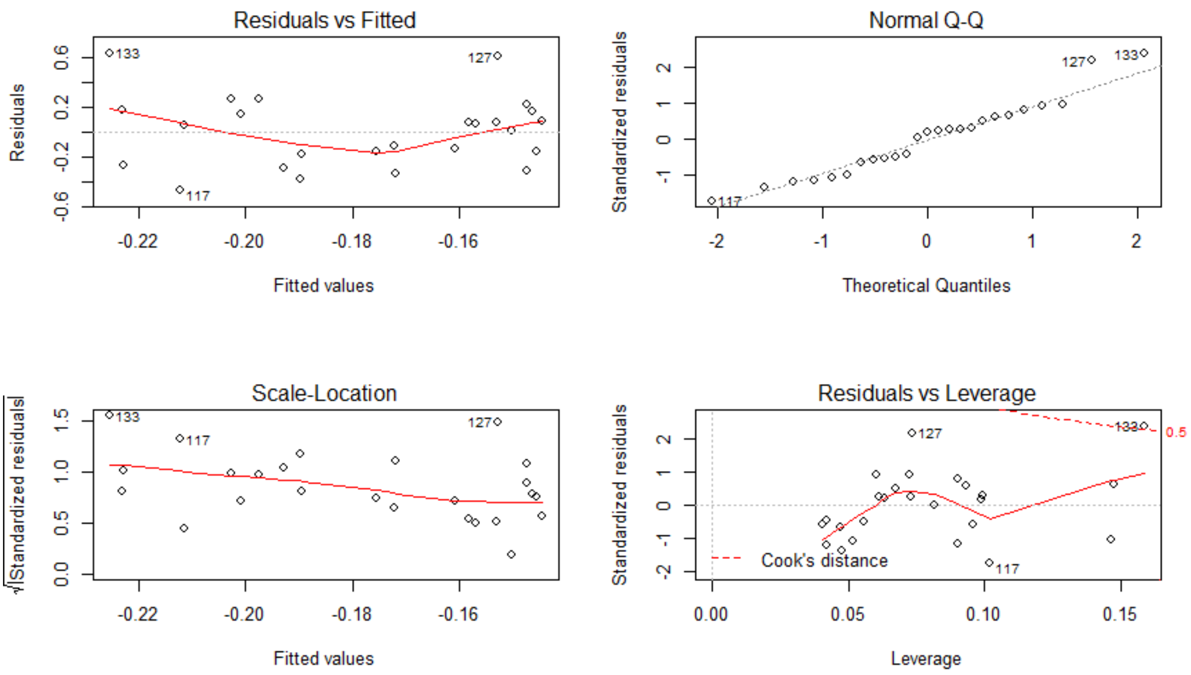


```
 Multiple regression power calculation 

              u = 1
              v = 24
             f2 = 0.0100704
      sig.level = 0.01
          power = 0.01907551
```

NAOtro SSN (150 BCE - 2012 CE)

```
Call:
lm(formula = Trouet ~ SSN...0, data = NAOtro_SSN0)

Residuals:
     Min       1Q   Median       3Q      Max 
-2.84727 -0.89487  0.08796  0.72923  1.89768 

Coefficients:
             Estimate Std. Error t value Pr(>|t|)    
(Intercept)  1.066773   0.267920   3.982 0.000167 ***
SSN...0     -0.009953   0.008260  -1.205 0.232331    
---
Signif. codes:  0 ‘***’ 0.001 ‘**’ 0.01 ‘*’ 0.05 ‘.’ 0.1 ‘ ’ 1

Residual standard error: 1.147 on 69 degrees of freedom
  (146 observations deleted due to missingness)
Multiple R-squared:  0.02061,	Adjusted R-squared:  0.006415 
F-statistic: 1.452 on 1 and 69 DF,  p-value: 0.2323


Anova Table (Type II tests)

Response: Trouet
          Sum Sq Df F value Pr(>F)
SSN...0    1.909  1   1.452 0.2323
Residuals 90.717 69
```

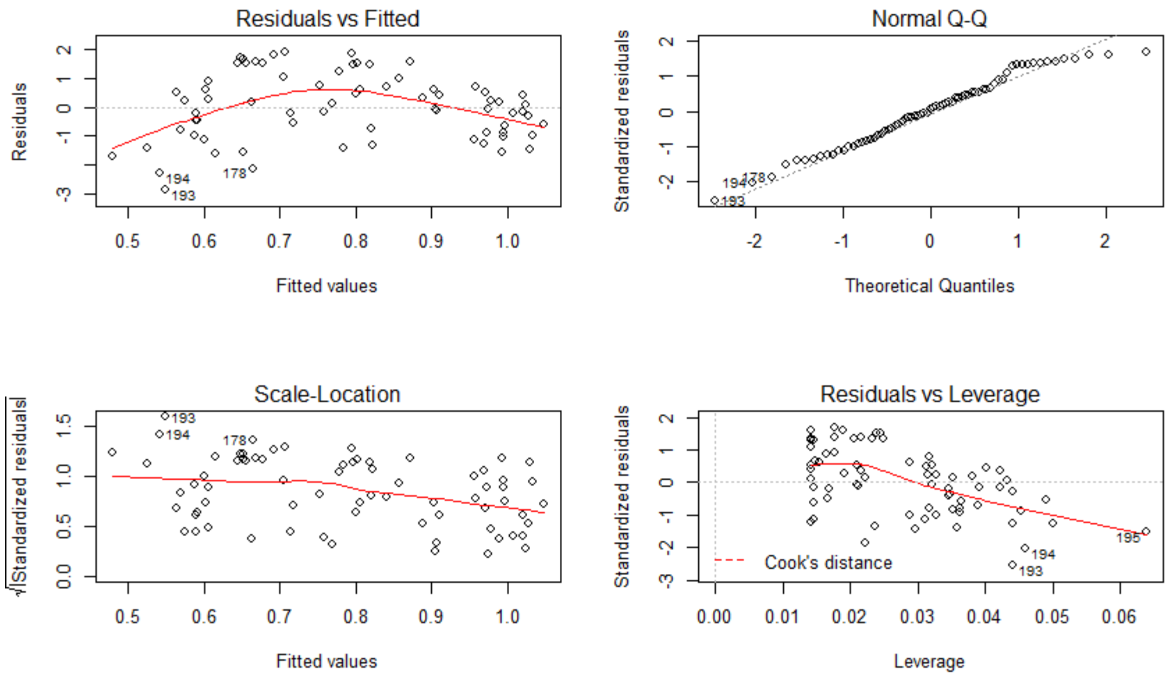


```
     Multiple regression power calculation 

              u = 1
              v = 70
             f2 = 0.02145046
      sig.level = 0.01
          power = 0.08663231
```

NAOtro SSN<42 (150 BCE - 2012 CE)

```
Call:
lm(formula = Trouet ~ SSN, data = NAOtro_SSN42)

Residuals:
    Min      1Q  Median      3Q     Max 
-2.9043 -0.6481  0.2030  0.7908  1.4844 

Coefficients:
            Estimate Std. Error t value Pr(>|t|)  
(Intercept)  0.53507    0.25972   2.060   0.0445 *
SSN          0.02274    0.01060   2.146   0.0367 *
---
Signif. codes:  0 '***' 0.001 '**' 0.01 '*' 0.05 '.' 0.1 ' ' 1

Residual standard error: 0.9862 on 51 degrees of freedom
  (82 observations deleted due to missingness)
Multiple R-squared:  0.08282,	Adjusted R-squared:  0.06484 
F-statistic: 4.605 on 1 and 51 DF,  p-value: 0.03665


Anova Table (Type II tests)

Response: Trouet
          Sum Sq Df F value  Pr(>F)  
SSN        4.479  1  4.6055 0.03665 *
Residuals 49.600 51                  
---
Signif. codes:  0 '***' 0.001 '**' 0.01 '*' 0.05 '.' 0.1 ' ' 1
```

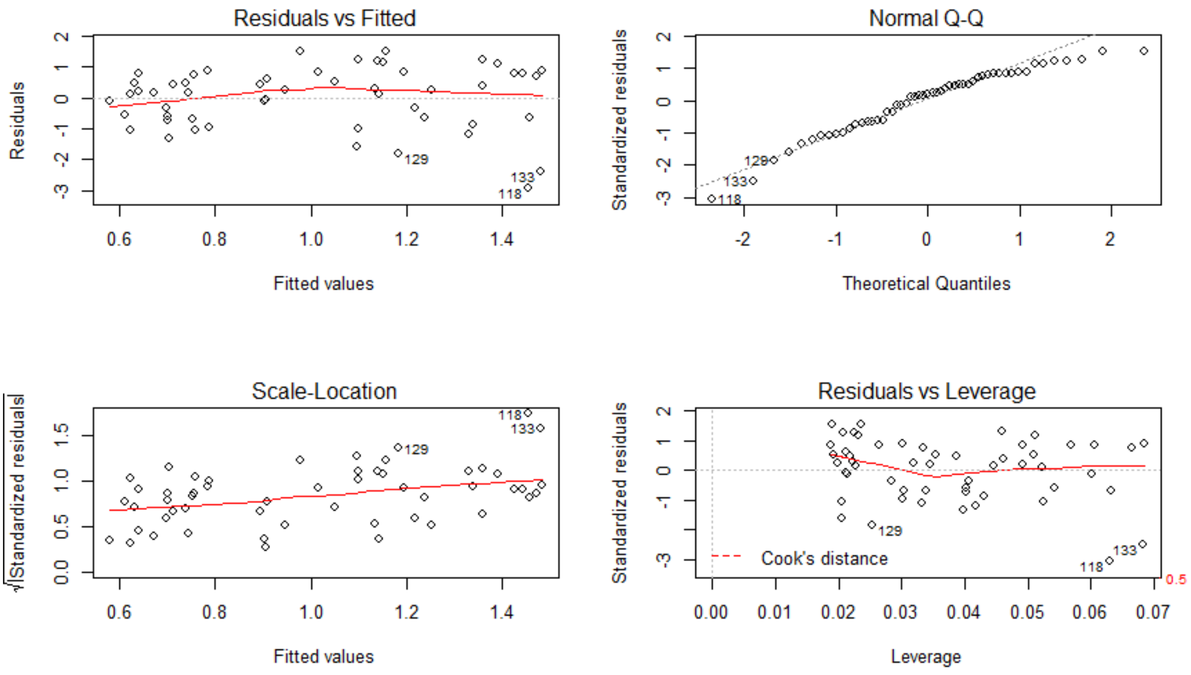


```
Multiple regression power calculation 

              u = 1
              v = 52
             f2 = 0.09029852
      sig.level = 0.01
          power = 0.3307785
```

NAOmor SSN (150 BCE - 2012 CE)

```
Call:
lm(formula = Morocco ~ SSN...0, data = NAOmor_SSN0)

Residuals:
    Min      1Q  Median      3Q     Max 
-1.3693 -0.4855  0.0865  0.4943  1.5028 

Coefficients:
             Estimate Std. Error t value Pr(>|t|)
(Intercept)  0.110784   0.159801   0.693    0.490
SSN...0     -0.004489   0.004927  -0.911    0.365

Residual standard error: 0.6839 on 69 degrees of freedom
  (146 observations deleted due to missingness)
Multiple R-squared:  0.01189,	Adjusted R-squared:  -0.00243 
F-statistic: 0.8303 on 1 and 69 DF,  p-value: 0.3654


Anova Table (Type II tests)

Response: Morocco
          Sum Sq Df F value Pr(>F)
SSN...0    0.388  1  0.8303 0.3654
Residuals 32.273 69
```

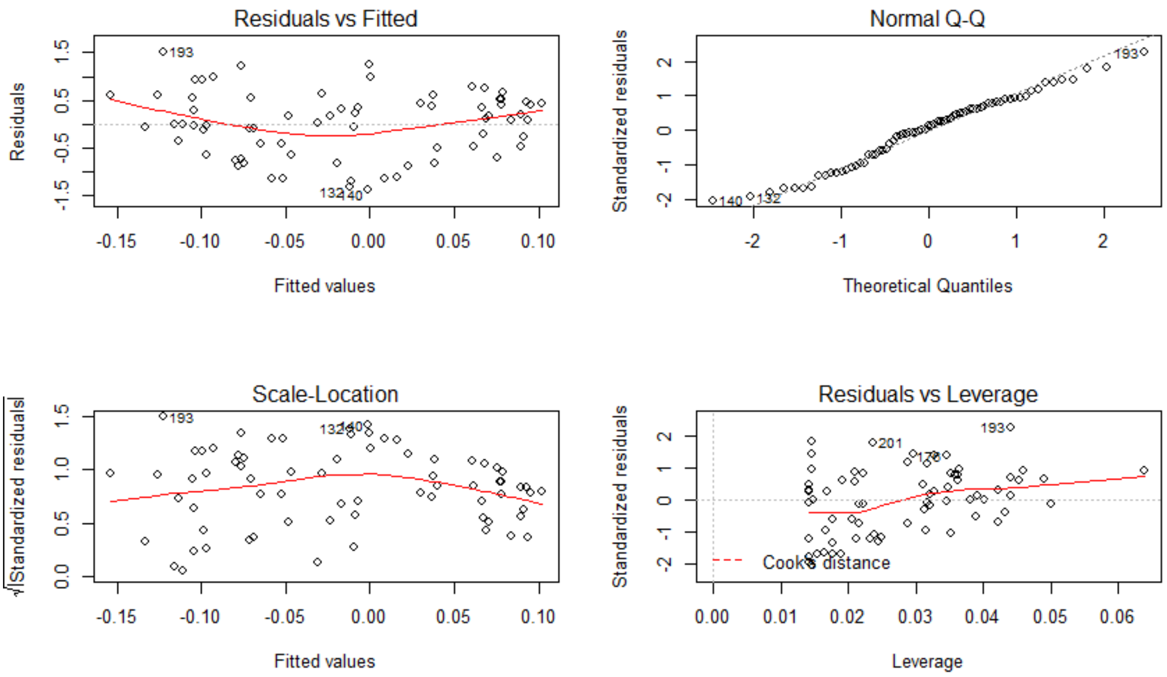


```
     Multiple regression power calculation 

              u = 1
              v = 70
             f2 = 0.01214575
      sig.level = 0.01
          power = 0.04843748
```

NAOmor SSN<42 (150 BCE - 2012 CE)

```
Call:
lm(formula = Morocco ~ SSN, data = NAOmor_SSN42)

Residuals:
     Min       1Q   Median       3Q      Max 
-1.25129 -0.49216  0.09259  0.41822  1.55760 

Coefficients:
             Estimate Std. Error t value Pr(>|t|)  
(Intercept)  0.324291   0.175117   1.852   0.0698 .
SSN         -0.017834   0.007144  -2.496   0.0158 *
---
Signif. codes:  0 ‘***’ 0.001 ‘**’ 0.01 ‘*’ 0.05 ‘.’ 0.1 ‘ ’ 1

Residual standard error: 0.6649 on 51 degrees of freedom
  (82 observations deleted due to missingness)
Multiple R-squared:  0.1089,	Adjusted R-squared:  0.09141 
F-statistic: 6.231 on 1 and 51 DF,  p-value: 0.01582


Anova Table (Type II tests)

Response: Morocco
          Sum Sq Df F value  Pr(>F)  
SSN       2.7551  1  6.2313 0.01582 *
Residuals 22.5491 51                  
---
Signif. codes:  0 ‘***’ 0.001 ‘**’ 0.01 ‘*’ 0.05 ‘.’ 0.1 ‘ ’ 1
```

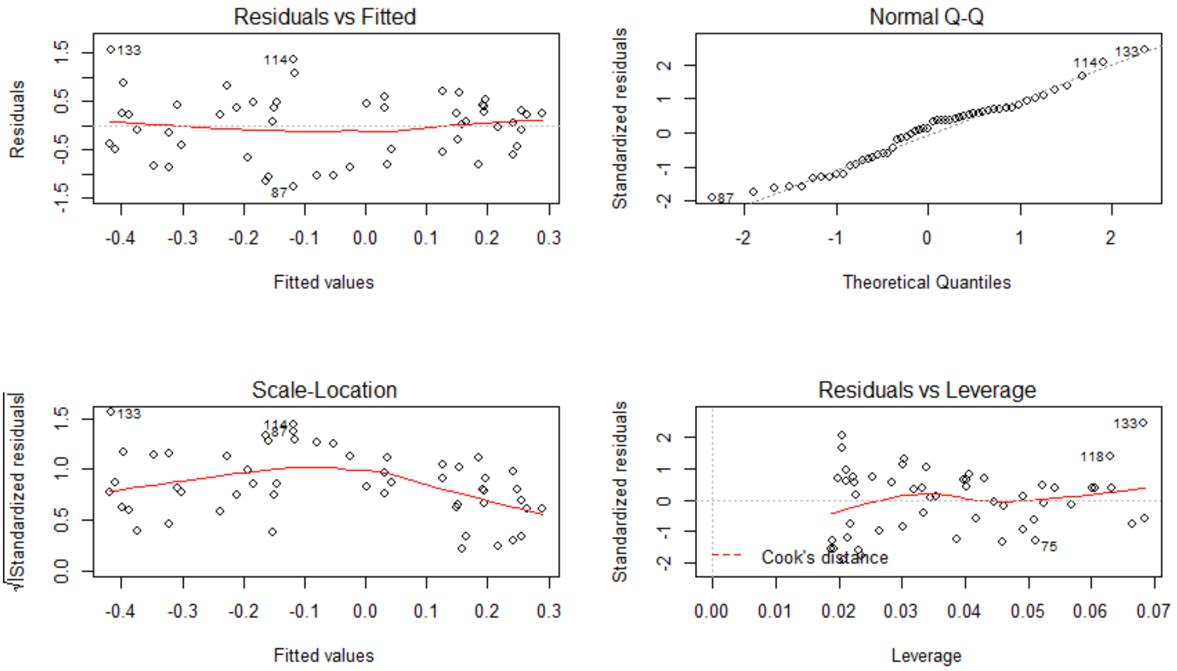


```
 Multiple regression power calculation 

              u = 1
              v = 52
             f2 = 0.1222085
      sig.level = 0.01
          power = 0.4646412
```

NAOols SSN (150 BCE - 2012 CE)

```
Call:
lm(formula = Olsen ~ SSN...0, data = NAOols_SSN0)

Residuals:
    Min      1Q  Median      3Q     Max 
-2.6146 -0.4544  0.1717  0.5729  1.2383 

Coefficients:
             Estimate Std. Error t value Pr(>|t|)    
(Intercept)  1.590234   0.194622   8.171    7e-13 ***
SSN...0     -0.005446   0.005271  -1.033    0.304    
---
Signif. codes:  0 ‘***’ 0.001 ‘**’ 0.01 ‘*’ 0.05 ‘.’ 0.1 ‘ ’ 1

Residual standard error: 0.7564 on 106 degrees of freedom
  (109 observations deleted due to missingness)
Multiple R-squared:  0.00997,  Adjusted R-squared:  0.0006296 
F-statistic: 1.067 on 1 and 106 DF,  p-value: 0.3039


Anova Table (Type II tests)

Response: Olsen
          Sum Sq  Df F value Pr(>F)
SSN...0    0.611   1  1.0674 0.3039
Residuals 60.649 106
```

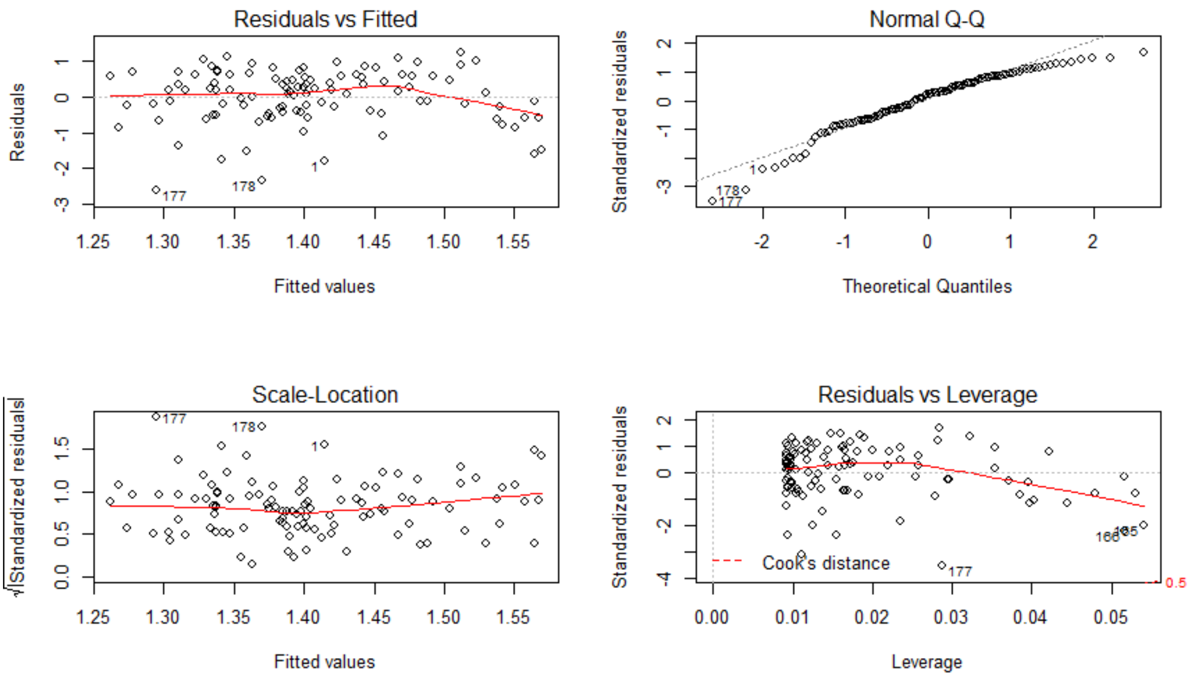


```
     Multiple regression power calculation 

              u = 1
              v = 107
             f2 = 0.01010101
      sig.level = 0.01
          power = 0.06160541
```

NAOols SSN<42 (150 BCE - 2012 CE)

```
Call:
lm(formula = Olsen ~ SSN, data = NAOols_SSN42)

Residuals:
    Min      1Q  Median      3Q     Max 
-2.4482 -0.4664  0.1126  0.5024  1.2955 

Coefficients:
             Estimate Std. Error t value Pr(>|t|)    
(Intercept) 1.4413050  0.2224131   6.480 9.89e-09 ***
SSN         0.0009129  0.0075285   0.121    0.904    
---
Signif. codes:  0 ‘***’ 0.001 ‘**’ 0.01 ‘*’ 0.05 ‘.’ 0.1 ‘ ’ 1

Residual standard error: 0.7097 on 72 degrees of freedom
  (61 observations deleted due to missingness)
Multiple R-squared:  0.0002042,	Adjusted R-squared:  -0.01368 
F-statistic: 0.0147 on 1 and 72 DF,  p-value: 0.9038


Anova Table (Type II tests)

Response: Olsen
          Sum Sq Df F value Pr(>F)
SSN        0.007  1  0.0147 0.9038
Residuals 36.269 72
```

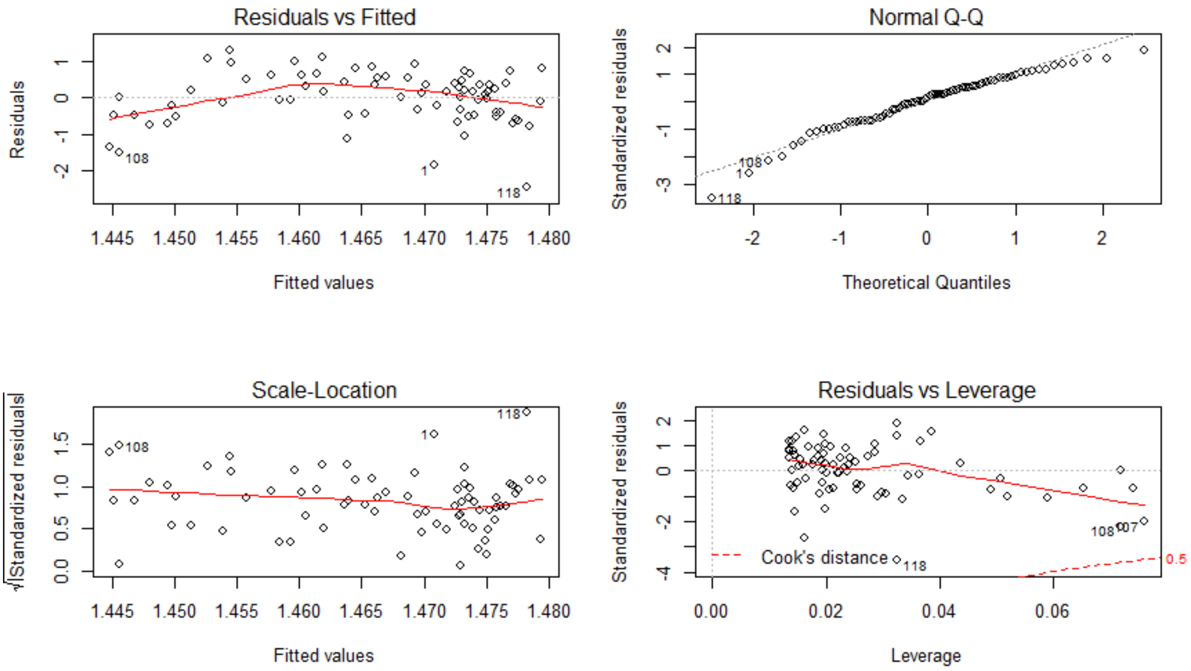


```
 Multiple regression power calculation 

              u = 1
              v = 72
             f2 = 0.0002042417
      sig.level = 0.01
          power = 0.01053998
```

NAOort SSN (150 BCE - 2012 CE)

```
Call:
lm(formula = Ortega ~ SSN...0, data = NAOort_SSN0)

Residuals:
    Min      1Q  Median      3Q     Max 
-1.8858 -0.1951  0.0394  0.2311  1.1459 

Coefficients:
             Estimate Std. Error t value Pr(>|t|)
(Intercept)  0.017472   0.121285   0.144    0.886
SSN...0     -0.004641   0.003739  -1.241    0.219

Residual standard error: 0.5191 on 69 degrees of freedom
  (146 observations deleted due to missingness)
Multiple R-squared:  0.02184,	Adjusted R-squared:  0.007659 
F-statistic:  1.54 on 1 and 69 DF,  p-value: 0.2188


Anova Table (Type II tests)

Response: Ortega
          Sum Sq Df F value Pr(>F)
SSN...0    0.415  1  1.5402 0.2188
Residuals 18.590 69
```

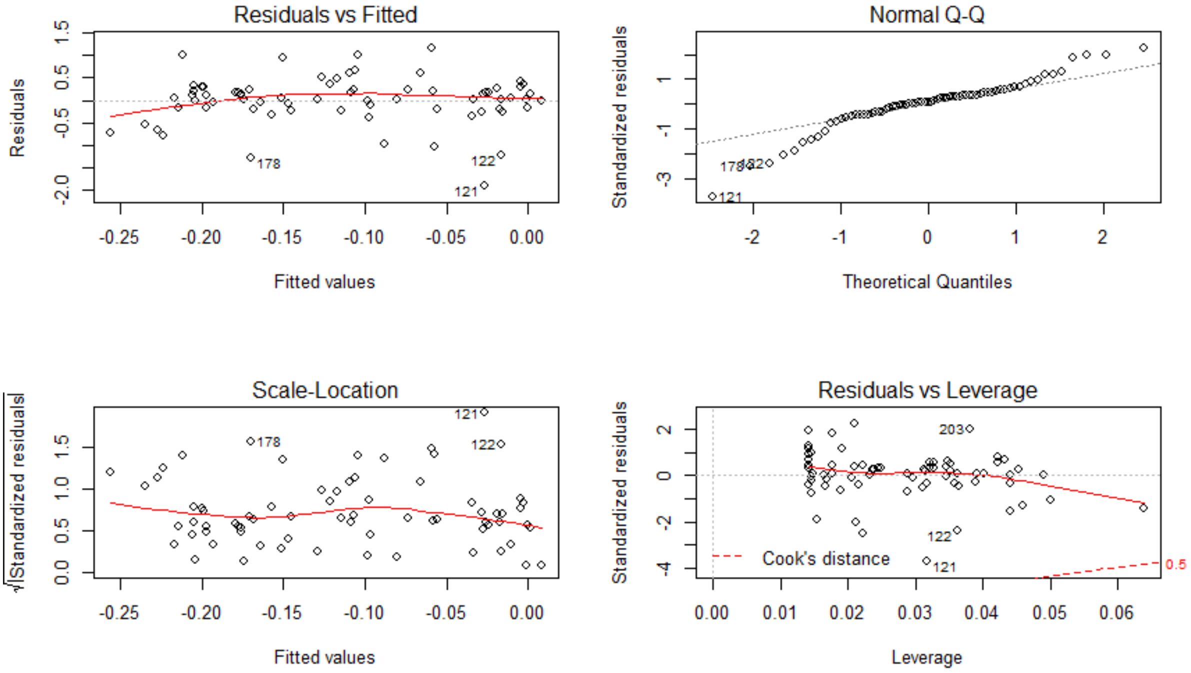


```
     Multiple regression power calculation 

              u = 1
              v = 70
             f2 = 0.02249489
      sig.level = 0.01
          power = 0.05539714
```

NAOort SSN<42 (150 BCE – 2012 CE)

```
Call:
lm(formula = Ortega ~ SSN, data = NAOort_SSN42)

Residuals:
     Min       1Q   Median       3Q      Max 
-1.85446 -0.19662  0.05378  0.21694  1.15357 

Coefficients:
             Estimate Std. Error t value Pr(>|t|)
(Intercept) -0.046310   0.143511  -0.323    0.748
SSN         -0.001231   0.005855  -0.210    0.834

Residual standard error: 0.5449 on 51 degrees of freedom
  (82 observations deleted due to missingness)
Multiple R-squared:  0.0008656,	Adjusted R-squared:  -0.01873 
F-statistic: 0.04418 on 1 and 51 DF,  p-value: 0.8344


Anova Table (Type II tests)

Response: Ortega
           Sum Sq Df F value Pr(>F)
SSN        0.0131  1  0.0442 0.8344
Residuals 15.1440 51
```

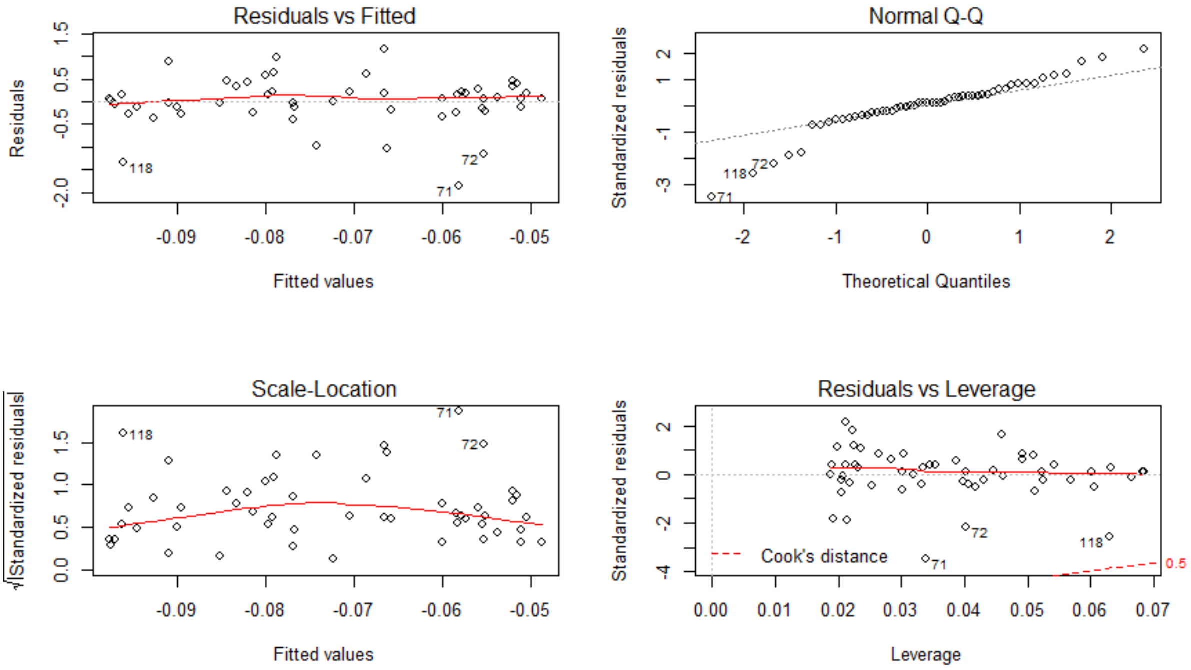


```
  Multiple regression power calculation 

              u = 1
              v = 52
             f2 = 0.0008663499
      sig.level = 0.01
          power = 0.01165672
```

NAObak SSN (150 BCE - 2012 CE)

```
Call:
lm(formula = Baker ~ SSN, data = NAObak_SSN0)

Residuals:
     Min       1Q   Median       3Q      Max 
-2.23912 -0.44079  0.09249  0.49029  1.24714 

Coefficients:
             Estimate Std. Error t value Pr(>|t|)
(Intercept) -0.033900   0.119482  -0.284    0.777
V1           0.004016   0.003228   1.244    0.215

Residual standard error: 0.6864 on 201 degrees of freedom
Multiple R-squared:  0.00764,  Adjusted R-squared:  0.002703 
F-statistic: 1.547 on 1 and 201 DF,  p-value: 0.215


Anova Table (Type II tests)

Response: Baker
          Sum Sq  Df F value Pr(>F)
SSN        0.729   1  1.5475  0.215
Residuals 94.698 201
```

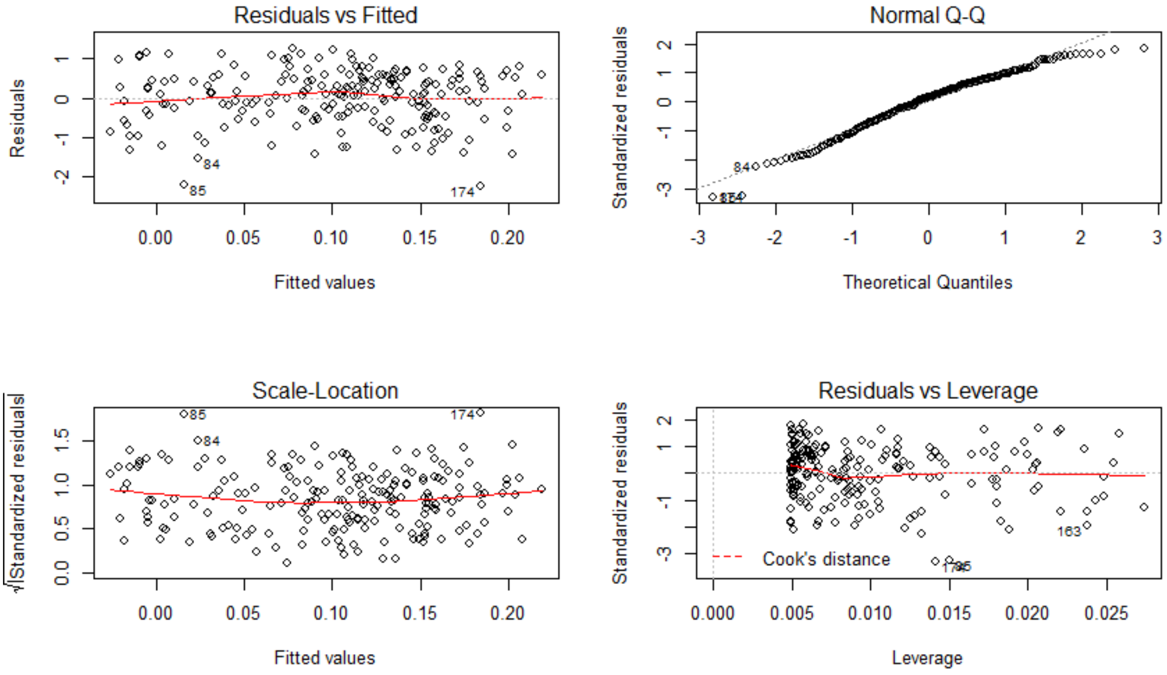


```
 Multiple regression power calculation 

              u = 1
              v = 202
             f2 = 0.008064516
      sig.level = 0.01
          power = 0.09622874
```

NAObak SSN<42 (150 BCE - 2012 CE)

```
Call:
lm(formula = Baker ~ SSN, data = NAObak_SSN42)

Residuals:
     Min       1Q   Median       3Q      Max 
-2.16252 -0.41363  0.03348  0.43182  1.24859 

Coefficients:
            Estimate Std. Error t value Pr(>|t|)   
(Intercept) -0.21160    0.13946  -1.517  0.13156   
SSN          0.01299    0.00488   2.662  0.00873 **
---
Signif. codes:  0 ‘***’ 0.001 ‘**’ 0.01 ‘*’ 0.05 ‘.’ 0.1 ‘ ’ 1

Residual standard error: 0.6671 on 133 degrees of freedom
Multiple R-squared:  0.05058,	Adjusted R-squared:  0.04344 
F-statistic: 7.085 on 1 and 133 DF,  p-value: 0.00873


Anova Table (Type II tests)

Response: Baker
          Sum Sq  Df F value  Pr(>F)   
SSN        3.153   1  7.0854 0.00873 **
Residuals 59.182 133                   
---
Signif. codes:  0 ‘***’ 0.001 ‘**’ 0.01 ‘*’ 0.05 ‘.’ 0.1 ‘ ’ 1
```

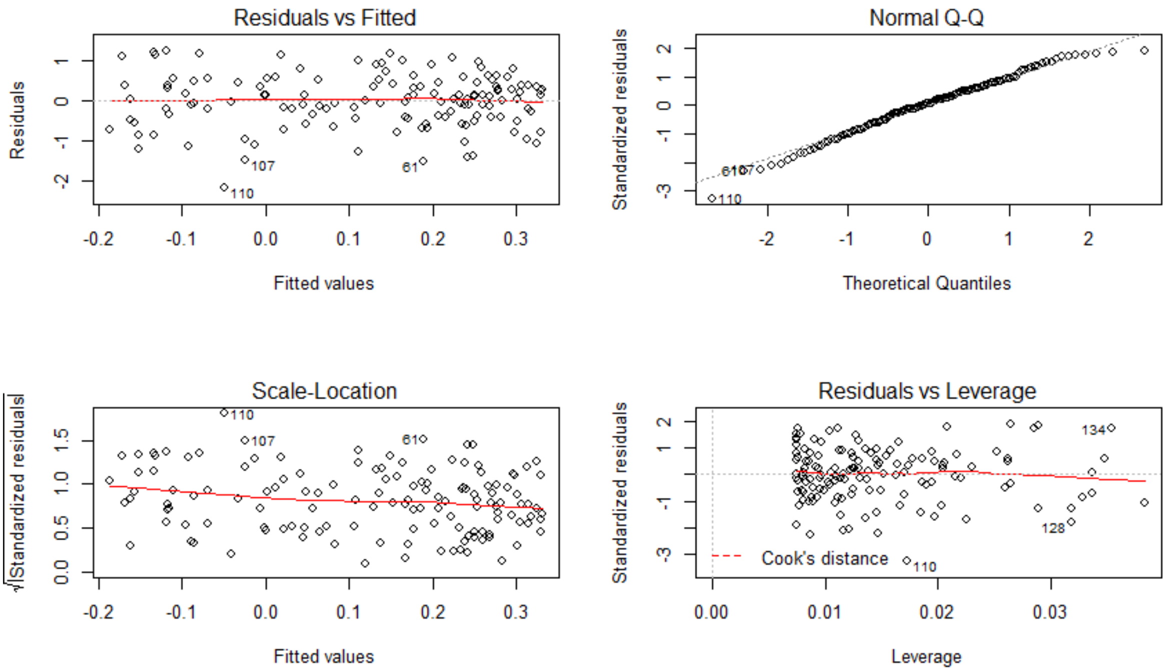


```
Multiple regression power calculation

              u = 1
              v = 133
             f2 = 0.05327463
      sig.level = 0.01
          power = 0.5289096
```

NAOfau SSN (150 BCE - 2012 CE)

```
Call:
lm(formula = Faust ~ SSN...0, data = NAOnor_SSN0)

Residuals:
     Min       1Q   Median       3Q      Max 
-1.41168 -0.32305  0.05402  0.27941  1.18627 

Coefficients:
             Estimate Std. Error t value Pr(>|t|)   
(Intercept) -0.280618   0.090263  -3.109  0.00218 **
SSN...0      0.006259   0.002405   2.603  0.01001 * 
---
Signif. codes:  0 ‘***’ 0.001 ‘**’ 0.01 ‘*’ 0.05 ‘.’ 0.1 ‘ ’ 1

Residual standard error: 0.4791 on 183 degrees of freedom
  (32 observations deleted due to missingness)
Multiple R-squared:  0.03569,  Adjusted R-squared:  0.03042 
F-statistic: 6.774 on 1 and 183 DF,  p-value: 0.01001


Anova Table (Type II tests)

Response: Faust
          Sum Sq  Df F value  Pr(>F)  
SSN...0    1.555   1  6.7736 0.01001 *
Residuals 42.010 183                  
---
Signif. codes:  0 ‘***’ 0.001 ‘**’ 0.01 ‘*’ 0.05 ‘.’ 0.1 ‘ ’ 1
```

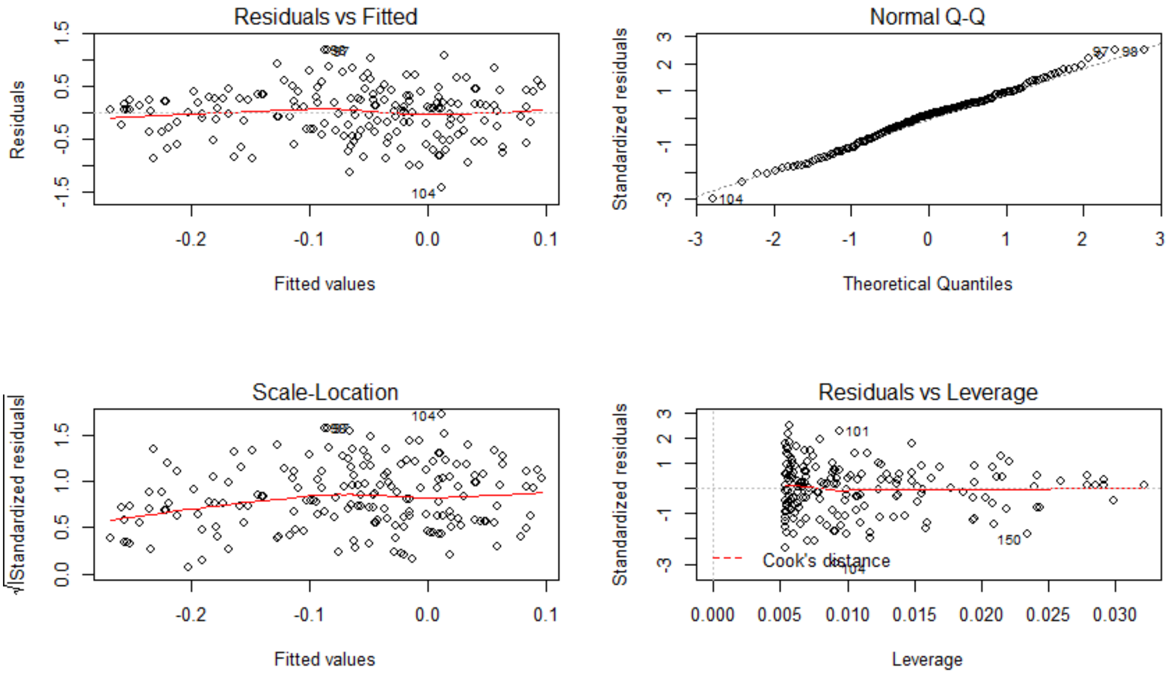


```
     Multiple regression power calculation 

              u = 1
              v = 184
             f2 = 0.0373444
      sig.level = 0.01
          power = 0.5143395
```

NAOfau SSN<42 (150 BCE - 2012 CE)

```
Call:
lm(formula = Faust ~ SSN, data = NAOnor_SSN42)

Residuals:
     Min       1Q   Median       3Q      Max 
-1.17832 -0.32671  0.03743  0.26753  1.15136 

Coefficients:
             Estimate Std. Error t value Pr(>|t|)   
(Intercept) -0.330663   0.103952  -3.181  0.00188 **
SSN          0.008995   0.003583   2.510  0.01341 * 
---
Signif. codes:  0 '***' 0.001 '**' 0.01 '*' 0.05 '.' 0.1 ' ' 1

Residual standard error: 0.4573 on 118 degrees of freedom
  (15 observations deleted due to missingness)
Multiple R-squared:  0.0507,	Adjusted R-squared:  0.04266 
F-statistic: 6.302 on 1 and 118 DF,  p-value: 0.01341


Anova Table (Type II tests)

Response: Faust
          Sum Sq  Df F value  Pr(>F)  
SSN        1.3178   1  6.3022 0.01341 *
Residuals 24.6739 118                  
---
Signif. codes:  0 '***' 0.001 '**' 0.01 '*' 0.05 '.' 0.1 ' ' 1
```

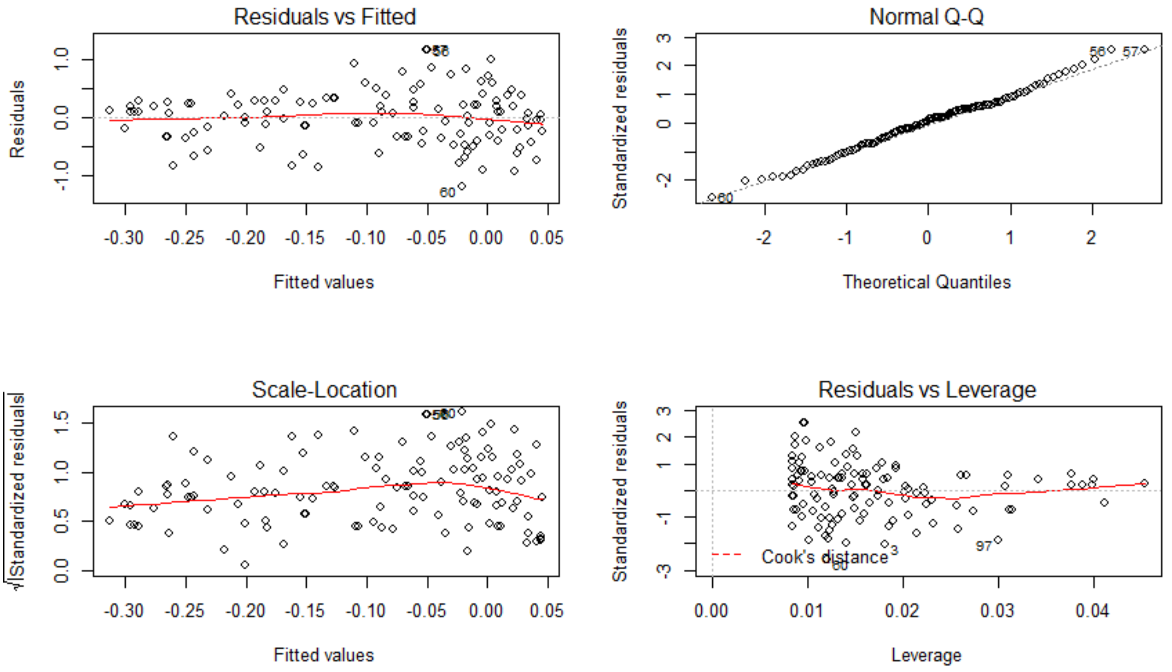


```
 Multiple regression power calculation 

              u = 1
              v = 119
             f2 = 0.05340777
      sig.level = 0.01
          power = 0.4724426
```

NAOsjo SSN (150 BCE - 2012 CE)

```
Call:
lm(formula = Sjolte ~ SSN, data = NAOsjo_SSN)

Residuals:
     Min       1Q   Median       3Q      Max 
-0.78249 -0.23016 -0.03674  0.28127  1.00645 

Coefficients:
             Estimate Std. Error t value Pr(>|t|)  
(Intercept) -0.115565   0.080806  -1.430   0.1579  
V1           0.004431   0.002618   1.692   0.0958 .
---
Signif. codes:  0 ‘***’ 0.001 ‘**’ 0.01 ‘*’ 0.05 ‘.’ 0.1 ‘ ’ 1

Residual standard error: 0.3592 on 59 degrees of freedom
  (3 observations deleted due to missingness)
Multiple R-squared:  0.0463,	Adjusted R-squared:  0.03014 
F-statistic: 2.864 on 1 and 59 DF,  p-value: 0.09584



Anova Table (Type II tests)

Response: Sjolte
          Sum Sq Df F value  Pr(>F)  
SSN        0.3695  1  2.8644 0.09584 .
Residuals 7.6119 59                  
---
Signif. codes:  0 ‘***’ 0.001 ‘**’ 0.01 ‘*’ 0.05 ‘.’ 0.1 ‘ ’ 1
```

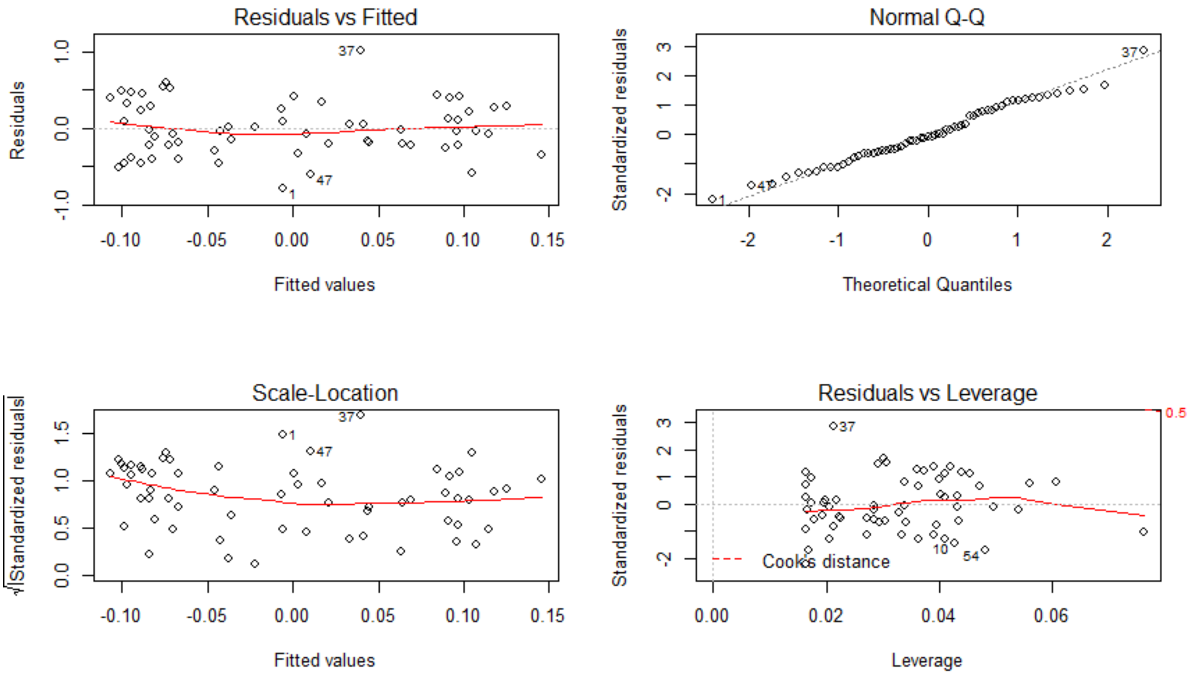


```
     Multiple regression power calculation 

              u = 1
              v = 60
             f2 = 0.04821803
      sig.level = 0.01
          power = 0.1855981
```

NAOsjo SSN<42 (150 BCE - 2012 CE)

```
Call:
lm(formula = Sjolte ~ SSN, data = NAOsjo_SSN42)

Residuals:
    Min      1Q  Median      3Q     Max 
-0.7522 -0.2392 -0.0354  0.2811  1.0598 

Coefficients:
             Estimate Std. Error t value Pr(>|t|)
(Intercept) -0.089417   0.096474  -0.927    0.359
V1           0.002159   0.004517   0.478    0.635

Residual standard error: 0.3763 on 44 degrees of freedom
  (3 observations deleted due to missingness)
Multiple R-squared:  0.005166,	Adjusted R-squared:  -0.01744 
F-statistic: 0.2285 on 1 and 44 DF,  p-value: 0.635


Anova Table (Type II tests)

Response: Sjolte
          Sum Sq Df F value Pr(>F)
SSN       0.0324  1  0.2285  0.635
Residuals 6.2319 44
```

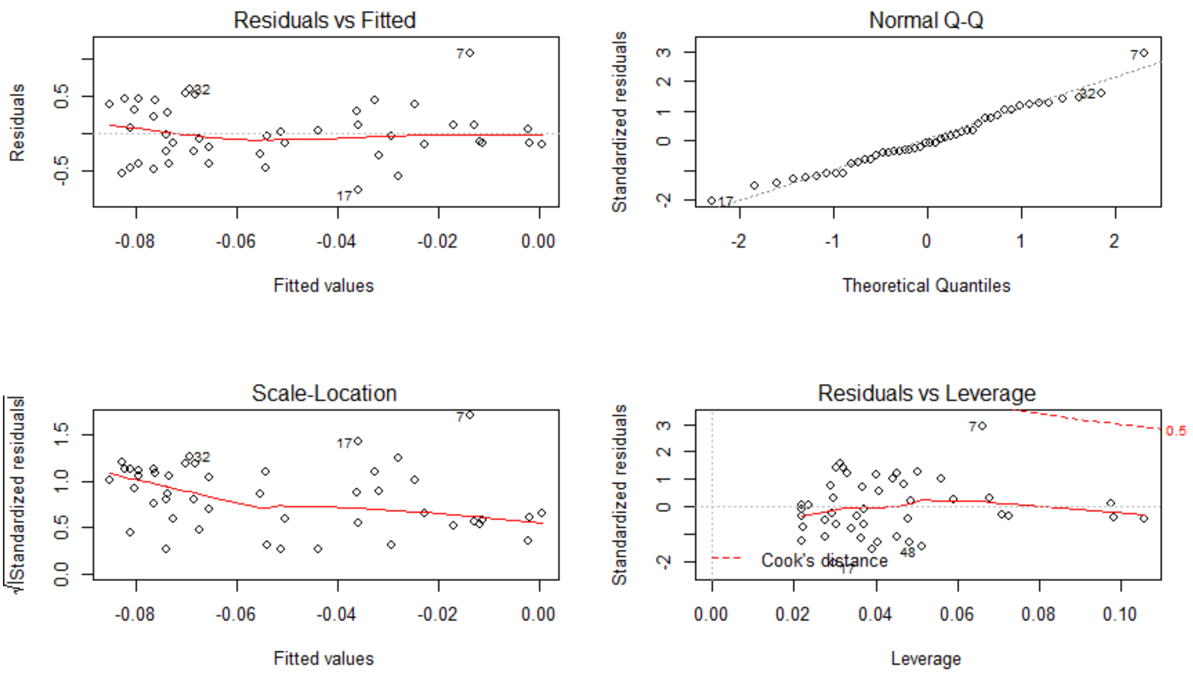


```
Multiple regression power calculation

              u = 1
              v = 45
             f2 = 0.005192826
      sig.level = 0.01
          power = 0.01901635
```

NAOcook SSN (150 BCE - 2012 CE)

```
Call:
lm(formula = Cook ~ SSN, data = NAOcook_SSN0)

Residuals:
     Min       1Q   Median       3Q      Max 
-0.71932 -0.26953  0.05933  0.20618  0.73210 

Coefficients:
             Estimate Std. Error t value Pr(>|t|)    
(Intercept)  0.380696   0.065138   5.844 7.64e-08 ***
V1          -0.001632   0.002003  -0.815    0.417    
---
Signif. codes:  0 ‘***’ 0.001 ‘**’ 0.01 ‘*’ 0.05 ‘.’ 0.1 ‘ ’ 1

Residual standard error: 0.3187 on 92 degrees of freedom
Multiple R-squared:  0.007162,	Adjusted R-squared:  -0.00363 
F-statistic: 0.6636 on 1 and 92 DF,  p-value: 0.4174


Anova Table (Type II tests)

Response: Cook
          Sum Sq Df F value Pr(>F)
SSN       0.0674  1  0.6636 0.4174
Residuals 9.3425 92
```

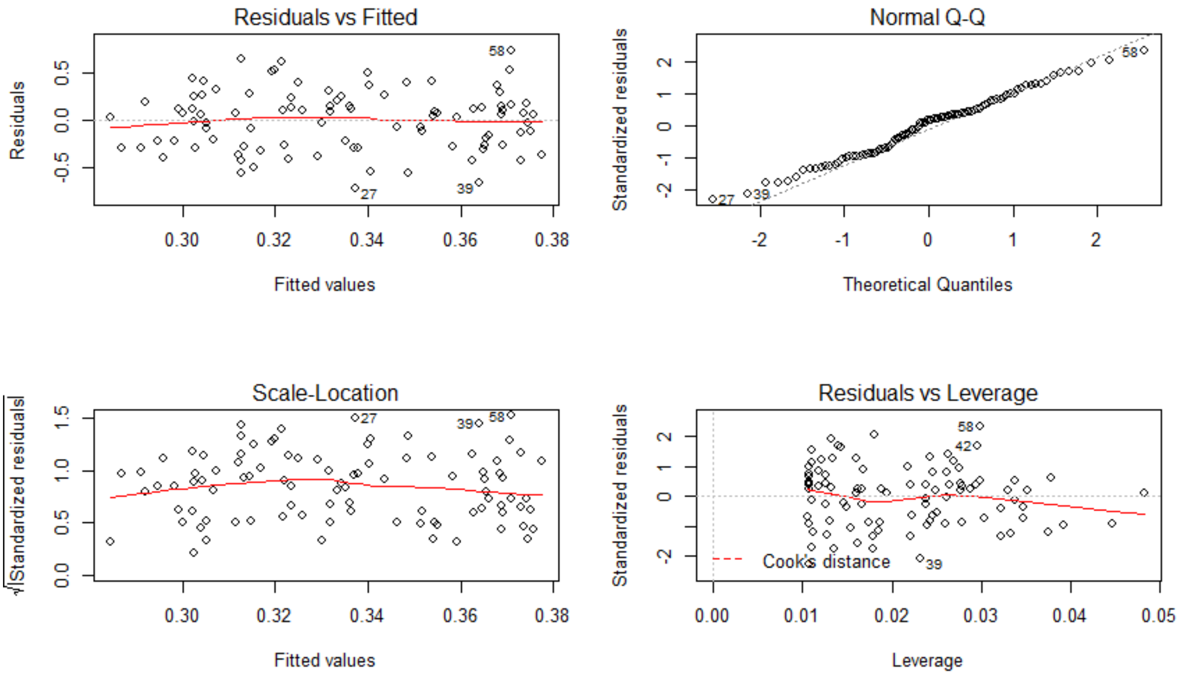


```
Multiple regression power calculation

              u = 1
              v = 93
             f2 = 0.007049345
      sig.level = 0.01
          power = 0.03856356
```

NAOcook SSN<42 (150 BCE - 2012 CE)

```
Call:
lm(formula = Cook ~ SSN, data = NAOcook_SSN42)

Residuals:
     Min       1Q   Median       3Q      Max 
-0.72379 -0.27646  0.07016  0.21102  0.74097 

Coefficients:
              Estimate Std. Error t value Pr(>|t|)    
(Intercept)  0.3679313  0.0785120   4.686 1.35e-05 ***
V1          -0.0009836  0.0031664  -0.311    0.757    
---
Signif. codes:  0 ‘***’ 0.001 ‘**’ 0.01 ‘*’ 0.05 ‘.’ 0.1 ‘ ’ 1

Residual standard error: 0.3396 on 69 degrees of freedom
Multiple R-squared:  0.001397, Adjusted R-squared:  -0.01308 
F-statistic: 0.09651 on 1 and 69 DF,  p-value: 0.757

Anova Table (Type II tests)

Response: Cook
          Sum Sq Df F value Pr(>F)
SSN        0.0111  1  0.0965  0.757
Residuals 7.9576 69
```

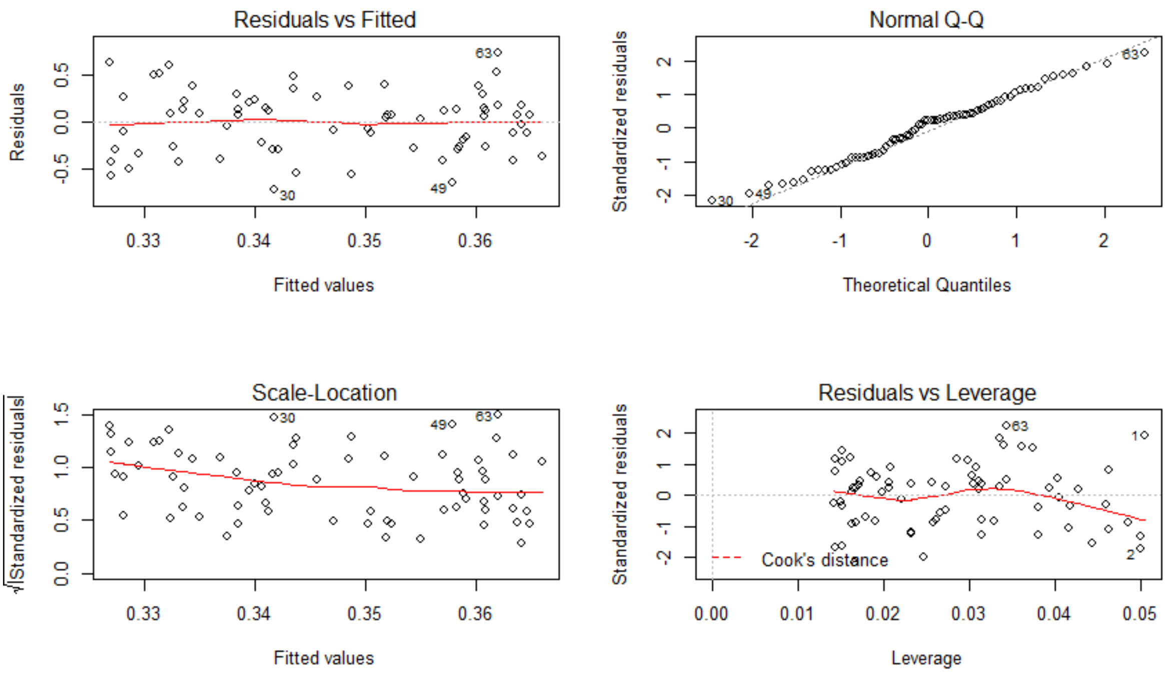


```
Multiple regression power calculation

              u = 1
              v = 70
             f2 = 0.001398954
      sig.level = 0.01
          power = 0.01368165
```

Breakpoint analysis of the NAO and sunspot number reconstruction relationship

	SSN	NAO
1	2.01	-0.70
2	3.41	-1.01
3	3.85	-0.84
4	3.89	-1.55
5	4.25	-0.61
6	4.68	-0.82
7	4.68	-1.23
8	6.00	-1.05
9	7.17	-0.42
10	7.29	-0.59
11	7.31	-0.68
12	7.43	-0.68
13	7.79	0.27
14	8.96	0.47
15	9.26	-0.76
16	9.53	-1.41
17	9.70	0.22
18	9.79	-0.82
19	11.04	-0.14
20	11.05	-0.49
21	12.48	-0.03
22	13.20	-0.62
23	13.79	-0.29
24	14.42	0.24
25	14.46	0.77
26	15.28	-0.26
27	15.82	-0.15
28	16.17	0.32
29	16.36	-0.38
30	16.46	-0.34
31	17.26	0.14
32	17.99	0.13
33	18.03	-0.34
34	18.75	-1.13
35	19.72	0.36
36	19.96	0.20
37	20.05	0.94
38	20.69	-0.36
39	21.12	-0.32
40	21.98	1.05
41	22.52	-0.10
42	22.67	0.13
43	24.49	1.19
44	24.64	-0.80
45	24.82	-0.32
46	25.47	-0.27
47	26.27	0.06
48	26.58	1.68
49	26.74	-1.08
50	26.89	0.42
51	27.35	0.94
52	28.05	0.33
53	28.43	-0.63
54	29.16	0.96
55	29.39	-0.10
56	29.92	0.11
57	29.96	0.34
58	30.01	-0.69
59	30.60	0.60
60	30.67	0.97
61	30.91	-0.59
62	31.05	0.31
63	31.26	1.30
64	31.57	0.68
65	32.29	0.90
66	32.54	0.64
67	32.85	-0.10
68	33.45	0.39
69	33.65	0.90
70	34.15	-0.81
71	34.23	1.81
72	34.35	0.23
73	34.55	-0.57
74	34.62	0.47
75	34.63	1.35
76	34.76	0.83
77	34.94	0.93
78	34.96	-0.04
79	35.01	0.42
80	35.40	-0.43
81	35.50	0.25
82	35.57	2.09
83	35.78	1.69
84	35.81	1.45
85	36.26	-0.52
86	36.36	0.69
87	36.79	-0.15
88	36.93	-0.13
89	36.98	0.19
90	37.08	0.21
91	37.19	0.03
92	37.62	0.75
93	37.64	0.44
94	37.75	1.26
95	37.76	0.37
96	37.93	1.33
97	38.09	0.43
98	38.58	0.79
99	39.03	0.23
100	39.18	-0.23
101	39.45	0.66
102	39.46	1.69
103	39.71	1.18
104	39.93	0.29
105	40.45	0.33
106	40.51	-0.04
107	40.54	0.04
108	40.73	1.43
109	41.22	-0.15
110	41.23	1.40
111	41.60	-0.20
112	41.63	0.49
113	41.69	1.48
114	41.73	0.65
115	42.06	0.74
116	42.12	0.67
**117**	**42.37**	**1.33 BP**
118	42.40	0.19
119	42.42	0.30
120	42.52	0.03
121	42.83	0.10
122	43.20	0.31
123	43.68	-0.42
124	43.97	0.34
125	44.65	0.41
126	44.66	1.32
127	44.92	0.46
128	45.26	-0.72
129	45.58	-0.23
130	45.64	-0.35
131	46.23	0.06
132	46.30	-0.13
133	46.34	0.14
134	46.41	0.08
135	46.43	0.00
136	46.47	0.04
137	46.48	1.35
138	46.62	0.38
139	46.70	0.13
140	46.85	-0.05
141	46.90	-0.82
142	47.04	0.95
143	47.11	-0.26
144	47.38	0.27
145	47.82	-0.34
146	47.87	-0.40
147	47.92	-0.36
148	48.03	-0.21
149	48.12	0.28
150	48.91	0.00
151	49.05	-0.31
152	49.11	1.46
153	49.47	0.14
154	49.87	0.14
155	50.12	0.44
156	50.40	-0.21
157	51.29	0.79
158	51.37	0.36
159	51.38	0.47
160	51.39	0.93
161	52.03	-0.83
162	52.45	0.64
163	52.69	0.22
164	52.73	-0.20
165	53.85	0.54
166	53.87	0.04
167	54.29	-0.39
168	54.30	0.53
169	54.60	0.35
170	54.96	0.04
171	54.97	0.46
172	57.45	-0.50
173	58.16	0.01
174	58.17	-0.89
175	58.23	-0.09
176	58.91	0.34
177	59.27	1.09
178	59.82	0.81
179	60.26	0.10

```
Optimal 2-segment partition:

Call:
breakpoints.formula
(formula = NAO ~ SSN, h=3
data = NAOSSN_Break_line)

Breakpoints at observation
number:
117

Corresponding to breakdates:
0.6536313
```

References

1 Sigl M, Winstrup M, McConnell JR, Welten KC, Plunkett G, Ludlow F, Büntgen U, Caffee M, Chellman N, Dahl-Jensen D, Fischer H, Kipfstuhl S, Kostick C, Maselli OJ, Mekhaldi F, Mulvaney R, Muscheler R, Pasteris DR, Pilcher JR, Salzer M, Schüpbach S, Steffensen JP, Vinther BM, Woodruff TE (2015) Timing and climate forcing of volcanic eruptions for the past 2,500 years. Nature 523:543–549

2 Sanchez-Cabeza, J.A., Masque, P., Ani-Ragolta, I., 1998. 210Pb and 210Po analysis in sediments and soils by microwave acid digestion. J. Radioanal. Nucl. Chem. 227, 19-22.

3 Krishnaswamy, S., Lal, D., Martin, J.M., Meybeck, M., 1971. Geochronology of lake sediments. Earth Planet. Sci. Lett. 11, 407-414.

4 Rull, V., Stansell, N.D., Montoya, E., Bezada, M., Abbott, M.B., 2010. Palynological signal of the younger dryas in the tropical Venezuelan Andes. Quat. Sci. Rev. 29, 3045-3056.

5 Stuiver, M., Reimer, P.J., 1993. Extended C-14 data base and revised Calib 3.0 C-14 age calibration program. Radiocarbon 35, 215-230.

6 Reimer, P.J., Bard, E., Bayliss, A., Beck, J.W., Blackwell, P.G., Ramsey, C.B., Buck, C.E., Cheng, H., Edwards, R.L., Friedrich, M., Grootes, P.M., 2013. INTCAL13 and MARINE13 radiocarbon age calibration curves 0-50,000 years cal BP. Radiocarbon 55, 1869-1887.

7 Blaauw, M., 2010. Methods and code for 'classical' age-modelling of radiocarbon sequences. Quat. Geochronol. 5, 512-518.

8 Sánchez-López, G., Hernández, A., Pla-Rabes, S., Trigo, R.M., Toro, M., Granados, I., Sáez, A., Masqué, P., Pueyo, J.J., Rubio-Inglés, M.J., Giralt, S., 2016. Climate reconstruction for the last two millennia in central Iberia: the role of East Atlantic (EA), North Atlantic Oscillation (NAO) and their interplay over the Iberian Peninsula. Quat. Sci. Rev. 149, 135–150.
